# “Even with one Man, but with many women, you can get cervical cancer”: Qualitative exploration of women’s perceptions and experiences of cervical cancer screening in Uganda: A community-based study

**DOI:** 10.1371/journal.pgph.0007256

**Published:** 2026-09-18

**Authors:** Robert M. Bulamba, Fred Nalugoda, Amanda P. Miller, Alex Daama, Godfrey Kigozi, Grace K. Nalwoga, Stephen Watya, Emmanuel Kyasanku, Resty Nakajugo, Anna Mia Ekström, Emma Menya, Violet Nkwanzi, Ndejjo Rawlance, Juliana Namutundu, Steven Mugamba, William Byansi, Jackline B. Nammanda, Gertrude Nakigozi

**Affiliations:** 1 Department of Epidemiology and Behavioral Research, Africa Medical and Behavioral Sciences Organization (AMBSO), Kampala Uganda; 2 Department of Epidemiology and Biostatistics, San Diego State University, San Diego, United States of America; 3 Department of Infectious Diseases, Karolinska Institute, Stocholm, Sweden; 4 Department of Social Work, College of Health Sciences, East Tennessee State University (ETSU), East Tennessee, United States of America; 5 Department of Disease Control and Environmental Health, Makerere University School of Public Health (MakSPH), Kampala Uganda; 6 Department of Social Work, School of Social work Boston College, Boston, United States of America; Durban University of Technology, SOUTH AFRICA

## Abstract

Cervical cancer is a leading cause of cancer-related morbidity and mortality among women in low-resource settings, with Uganda being disproportionately affected. Despite the availability of screening services in Uganda, uptake remains unacceptably low, yet population-level data on women’s perceptions and experienced barriers to screening remain limited. This hinders the development of targeted community-based interventions for cervical cancer screening in the country. To close this gap, we explored perceptions, attitudes, knowledge, and barriers to cervical cancer screening among women aged 25–65 years in the urban and rural areas of Wakiso District, Uganda. We conducted six focus group discussions - three in rural and three in urban with sixty women, and four key informant interviews with District health officers and healthcare providers in the urban and rural communities. Knowledge levels varied regarding cervical cancer risk factors, causes, signs, symptoms, and prevention. Participants in the focus group discussions linked cervical cancer to risky sexual behaviors, particularly involving multiple sexual partners or sexual intercourse with uncircumcised men, while some key informants said that some women believed that the disease was due to witchcraft. Concerns were also raised about family planning methods such as Intrauterine Devices (IUDs) and pills, which some participants believed to cause cervical cancer. Poor hygiene practices, including the use of unclean public toilets and poor menstrual hygiene, were also seen as risk factors. Myths and misconceptions about cervical cancer screening, such as fears of removal of the cervix during screening, were prevalent. Although some women had positive experiences with screening, concerns about pain, discomfort, and limited privacy during screening were commonly reported. While knowledge of cervical cancer exists among women in Wakiso District, significant misconceptions, fears, and systemic barriers impede screening uptake. Culturally sensitive health education and accessible, respectful screening services are critical to improving participation.

## Introduction

Cervical cancer is a deadly but preventable disease that disproportionately affects women in low- and middle-income countries (LMICs), accounting for more than 85% of global cervical cancer deaths [1]. In Uganda, cervical cancer is the leading cause of cancer-related deaths among women, with approximately 7000 new cases and more than 4,600 deaths annually [2]. Despite being preventable through timely screening and early treatment, cervical cancer continues to claim the lives of thousands of women annually due to limited access and poor utilization of preventive services [3]. The Ugandan Ministry of Health (MoH), in partnership with international partners, has introduced several interventions, including the integration of visual inspection with acetic acid (VIA) -a low cost cervical cancer screening method in which dilute acetic acid is applied to the cervix to highlight precancerous lesions, into routine reproductive health services and the rollout of Human Papillomavirus (HPV) vaccination among school-aged girls [4]. While these initiatives are commendable, implementation remains inconsistent, and national coverage of cervical cancer screening is estimated to be less than 30% [5].

This indicates that barriers beyond just service availability are impeding uptake. One major challenge is the ongoing gap in knowledge and awareness about cervical cancer in the community. Several studies have shown that many women, particularly in rural and peri-urban areas, are unaware of the risk factors, symptoms, and benefits of early detection [6–8]. Misconceptions are common, including beliefs that cervical cancer is caused by prolonged contraceptive use, poor hygiene, or witchcraft [9]. These beliefs are often influenced by informal social networks, peer discussions, and misinformation, which can discourage women from accessing screening services. Additionally, sociocultural factors play a significant role in women's health-seeking behaviors. In many Ugandan communities, gender norms and expectations influence access to reproductive health care. Women often need spousal permission to seek care, and disapproval from male partners is identified as a major barrier to cervical cancer screening [10]. In some cases, screening is linked to promiscuity, further reducing its acceptance among women.

Health system barriers such as long waiting times, inadequately trained personnel, stockouts of essential supplies, insufficient privacy during examinations, and poor healthcare workers’ attitudes also persist [11]. Women reported feeling disrespected or judged by health care personnel when attempting to access reproductive health services, which undermines trust and discourages follow-up visits [12]. Additionally, despite the availability of cervical cancer screening in some areas, the cost of related services such as transportation to the health facilities, consultation fees, diagnostic follow-up tests, treatment of detected lesions, and accessibility serve as barriers for many, particularly in underserved areas.

While quantitative studies have documented low screening rates, they often fail to explain the underlying reasons for this trend [13]. A qualitative approach allows for a deeper exploration of women’s beliefs, fears, and motivations, capturing the nuanced interplay between individual, interpersonal, and systemic factors. Although several previous qualitative studies have explored cervical cancer screening in Uganda, majority of these studies have been conducted among women attending health facilities, specific clinical populations, and a few conducted in communities have focused on limited geographical settings. Few studies have examined women’s perceptions from a population-based perspective while simultaneously incorporating healthcare providers’ experiences across both the rural and urban communities. By leveraging the APHS population cohort framework, our study provides broader community-level and context-specific insights into how knowledge, sociocultural norms, gender dynamics, and health system experiences interact to influence cervical cancer screening uptake among women to better inform population-level cervical cancer prevention interventions in Wakiso district and other similar areas in Uganda. The knowledge gap this study aimed to address lay in the limited understanding of how women interpret cervical cancer-related messages and how this influences their decision to utilize screening services. The study also sought to amplify the voices of women and healthcare providers, offering a platform for their insights to inform future interventions. The study adopts a comprehensive approach to examine the challenges and opportunities for increasing cervical cancer screening uptake in Uganda, by exploring individual-level factors (e.g., knowledge and perceptions), interpersonal and community influences (e.g., social norms and support), and health system barriers (e.g., accessibility and cost). By generating context-specific evidence from Wakiso District, the findings inform culturally sensitive, community-driven interventions to enhance cervical cancer prevention and control in Uganda, and contribute to national and global efforts aimed at eliminating cervical cancer as a public health threat by 2030 [4].

## Methods

### Ethical statement

The study was reviewed and approved by the Clark International University– Research Ethics Committee (CIU-REC) and registered with the Uganda National Council for Science and Technology (UNCST). Voluntary written informed consent was obtained from all study participants after the moderator or interviewer explained all the study aims, benefits, and potential risks in participating in this study, in accordance with the Declaration of Helsinki standard. No personally identifying information was included in audio recordings or transcripts, and participants were assigned unique identification codes or pseudonyms during transcription and analysis.

The anonymity of participants was diligently maintained throughout the research process by the use of numbers and the treatment of the data with confidentiality.

### Study area, design, and population

The study was conducted in Wakiso, the most populous district in Uganda, with a population of 3.4 million people [14], and the majority are women (51%), occupying approximately 1,884Km^2^ [15]. The major economic activities in the district include subsistence and commercial agriculture, fishing, and the operation of small and large trading and vending businesses in trading centres. This study used a qualitative descriptive design using content analysis to explore participants’ perspectives on cervical cancer screening in Uganda. The approach was chosen to allow for a systematic and data-driven identification of themes, while remaining close to participants’ accounts. The study was informed by an interpretivist perspective, acknowledging that meaning is co-constructed between participants and researchers. The study utilized focus group discussions (FGDs) with women aged 25–65 years who had lived in the study district for a minimum of six months. This age group was chosen because of their eligibility for cervical cancer screening services, have a higher risk for cervical cancer, and being targeted by the national programs for cervical cancer prevention in Uganda [16]. Key informant interviews (KIIs) were also conducted with members of the district health teams, including the district health officer and healthcare workers in both private and public healthcare facilities at different levels, such as nurses, medical officers, gynecologists, clinical officers, and midwives. Although this study was conducted in the same geographical setting as a previously published quantitative study [13], the two studies were independent. The present qualitative work used a different study design, sampling approach, and participant group, and did not draw on data or participants from the quantitative study. It was specifically designed to offer an in-depth understanding of the perceptions and barriers related to cervical cancer screening in the study area.

### Sampling

This study was nested within an ongoing Africa Medical and Behavioral Sciences Organization (AMBSO) Population Health Surveillance (APHS) Cohort study framework, being implemented in Wakiso and Hoima Districts in Uganda. The cohort profile has been described elsewhere [17]. Briefly, the APHS is an open longitudinal population-based cohort study established in 2018. The APHS is the first health and demographic surveillance system (HDSS) in Uganda to include urban sites in addition to semi-urban, rural, and fishing communities, and tracks health outcomes and their determinants in diverse communities. APHS focuses on under-researched and emerging public health issues in Uganda, including mental health, substance use, gender-based violence (GBV), violence against children, food insecurity, health behaviors, disability, communicable and non-communicable diseases (NCDs), and emerging diseases. Data collection from census activities and surveys target community types that exhibit considerable variation in risk behaviors and health conditions. As such, the cohort reflects the diversity of the Ugandan population and allows for stratification by district and community type.

The communities were selected to ensure a wide geographical coverage of the study area as well as to represent subpopulations of women in terms of rural-urban residence and socioeconomic status. Our qualitative study leveraged two (urban and rural) of the three Wakiso communities of APHS. We conducted a total of six (6) FGDs (3 in rural) and (3 in urban) settings, to allow exploration of context-specific differences in cervical cancer screening experiences and barriers, as these settings are known to differ in terms of healthcare access, infrastructure, and socio-cultural dynamics. The local leaders and the village health teams (VHTs, similar to community health workers) who were familiar with community members in the selected communities guided the identification and purposive recruitment of women participants eligible for interviews (e.g., age, residency, and relevance to cervical cancer experiences). The participants were then approached and invited to participate in the FGDs. To reduce potential selection bias, VHTs were guided to recruit participants with diverse characteristics, including variation in age, prior screening experience, and socioeconomic background. We ensured inclusion of participants from both rural and urban settings to capture a range of perspectives. Eligible participants were approached in person by trained research assistants, provided with detailed information about the study, and invited to participate voluntarily in the FGDs. Data collection continued iteratively until thematic saturation was reached, defined as the point at which no new themes emerged from the interviews. FGDs were conducted in a private setting to ensure that conversations could not be overheard.

Prior to the FGDs, participants were informed about the importance of maintaining confidentiality and were asked to respect the privacy of other group participants by not sharing discussion content outside the group. Written informed consent was obtained from all participants. The participants for the KIIs were purposively selected based on their technical knowledge, expertise, roles, and involvement in cervical cancer screening service delivery and decision-making relating to the provision of cervical cancer screening in the study district. Four (4) KIIs were conducted in the two communities (2 in rural and 2 in urban), and the numbers in both the FGDs and KIIs satisfied theoretical saturation [18], when no new ideas were emerging from participants. Additionally, participants’ data (audio recordings, demographics, and transcripts) were securely stored on password-protected devices, and only authorized research team members had access to the data. Despite these measures, complete anonymity cannot be guaranteed in focus group settings; however, all reasonable steps were taken to minimize risks to participants in the study.

### Data collection

We developed thematic guides for both the FGDs and KIIs based on previous literature [8,12,19] and pretested them among a similar study population (n = 4), before finalizing tools for data collection. Data collection for this present study was conducted over one week, from 25^th^ September to 2^nd^ October 2024. The FGD guide explored knowledge about cervical cancer and screening, attitudes, perceptions, barriers to participation, and strategies to improve women’s participation in cervical cancer screening services. The KII guide included questions on community perception and knowledge about cervical cancer and screening, health system capacity to perform cervical cancer screening, and opinions on measures to increase the utilization of screening services. The guides had probes and prompts to guide the research assistants during data collection. On average, each FGD comprised of 10 participants. FGDs were conducted by four (4) trained research assistants who are native Luganda speakers with vast experience conducting qualitative research. Each interview session was conducted by two (2) female research assistants, one of whom moderated and facilitated the discussion, while the other took notes and recorded the interviews. The FGDs were conducted in places identified by the research team in coordination with the local leaders and VHTs. Interview venues were carefully selected to ensure privacy, and all participants were encouraged to openly discuss their opinions. FGDs lasted for about 40–45 minutes, excluding 5–10 minutes for the informed consent process and rapport creation.

For the KIIs, when every key informant was identified, the research team scheduled appointments by phone, and interviews were conducted at time and place most convenient to the participant (most preferred their workplaces). KIIs lasted for about 30–35 minutes. All interviews were audio-recorded. The research assistants (RAs) comprised individuals with backgrounds in social work and social sciences, public health, clinical practice, and qualitative research, some of whom had prior experience working in the study setting. The teams’ professional training and prior assumptions about cervical cancer and screening may have influenced data collection and interpretation. However, to mitigate potential bias, we used standardized interview guides, engaged in regular team debrief sessions to reflect on emerging interpretations, and we applied iterative coding with multiple researchers reviewing transcripts. We then discussed and resolved discrepancies collaboratively. We also maintained analytic memos to document decision-making throughout the study implementation.

### Data management and analysis

The audio-labelled FGD recordings were transcribed verbatim from the local language (Luganda) into English and proofread several times by research assistants for accuracy. The transcripts were reviewed and emerging themes noted. All the researchers, RN (clinical nurse), EM (social scientist), JN (social scientist), and RB (Public health specialist) have experience in designing and conducting qualitative research. Three (3) of the researchers (EM, JN, and RB) are males, while RN is a female. Two researchers (EM and RB) independently developed the codebook for data analysis and described the coding tree, which was then reviewed and discussed with other researchers, and any differences were harmonized. Data was interpreted, coded, and then analyzed using content thematic analysis with the help of NVivo qualitative data management software. Direct quotations are presented in italics to highlight and support key findings. The consolidated Criteria for Reporting Qualitative Research (COREQ-32) checklist [20] guided the reporting of our results in this study. Data collection and analysis occurred concurrently to allow monitoring of emerging concepts across interviews. After familiarization with the transcripts, initial inductive codes were independently generated by two researchers before being compared and refined into a common coding framework. Codes were subsequently grouped into categories and broader themes through an iterative analytical process. Themes were then continuously reviewed and refined through regular discussions among the research team until consensus was achieved. Data collection ceased once successive focus group discussions and key informant interviews yielded no new codes or conceptual insights across the urban and rural settings, which indicated that thematic saturation had been achieved.

## Results

### Sociodemographic characteristics of the study participants

Of the 60 women who participated in the FGDs, median age was 38 years (IQR = 31–46 years), and most (38%) were 25–35 years old a (see Table 1). Majority (53%) were living in the urban area, married (63%), were involved in self-employment (business work), and were Catholic (47%). Over half of the participants had attained a secondary level education and above (53%).

**Table 1:** Socio-demographic characteristics of the FGD study participants.

Characteristics	Frequency (n = 60)	Proportion (%)
**Community Setting**		
Rural	28	47%
Urban	32	53%
**Age (Years)**, Median age = 38 years, IQR = 31–46 years		
25-35	23	38%
36-45	21	35%
46-65	16	27%
**Marital**		
Married	38	63%
Not married	17	28%
Widowed	5	8%
**Education Level**		
No education	2	3%
Primary	26	43%
Secondary and above	32	53%
**Employment Type**		
Business women	36	60%
Farmer	11	18%
Hair dresser	5	8%
Casual worker	3	6%
Others^**#**^	5	8%
**Religion**		
Anglicans	14	23%
Catholics	28	47%
Muslims	12	20%
Others*	6	10%

^**#**^Healthcare worker, casual workers, Housework in their own home, and None. ^*^Faith of Unity, Saved. Born again, Seventh Day Adventists, and None. *Seventh Day Adventists and Pentecostals.

### Thematic findings

The analysis of six focus group discussions and four key informant interviews generated four (4) major themes and several related subthemes describing women’s perceptions and experiences of cervical cancer screening. These themes included;

**Theme 1:** Knowledge and perceptions of cervical cancer and cervical cancer screening.
**Theme 2:** Cultural and Social barriers to cervical cancer screening.
**Theme 3:** Gender dynamics and power relations influencing screening decisions.
**Theme 4:** Health system-related barriers to cervical cancer screening.

Together, these themes demonstrated that women’s screening decisions are influenced by the interaction of individual beliefs, community norms, household power dynamics, and health system experiences rather than by knowledge alone.

#### Knowledge and perceptions of cervical cancer and cervical cancer screening.

Knowledge of cervical cancer and its screening was widespread among participants, although often shaped by myths and misconceptions. Many women demonstrated awareness that cervical cancer is primarily sexually transmitted, commonly linking it to men’s risky sexual behaviors, especially adultery and poor genital hygiene. Participants perceived that having multiple sexual partners, intercourse with uncircumcised men, engaging in sex during menstruation, or shortly after childbirth were major risk factors of cervical cancer. Some believed that even one’s monogamy could not protect them if their partner had unprotected sex with others:

*“Doctor, what I know, even if you have only one*

*man, but he has another woman, you can get cervical*

*cancer through him” [FGD Participant, aged*

*25-35 years].*

Misconceptions included beliefs that family planning methods, especially the intrauterine device (IUD), cause cervical cancer disease, linking its long-term use to rusting metal in the body.

*“I heard from my friends where I stay that the family planning*

*methods we use especially IUD causes cervical cancer. They even*

*gave me an example that if a metal can rust when placed on a mere*

*substance, how dangerous could it be in the human body?”*

Additionally, some women thought that halting menstruation using medication or engaging in sex during menstrual periods caused “blood clots” that could trigger cancer.

*“For me I think, for the women who get into their menstruation*

*period, then they use medicines that stops the blood from flowing*

*yet the blood is meant to flow. Each time you prevent the blood from*

*flowing out it creates blood clots, leading to cancer of the*

*cervix”[FGD Participant, aged 36-45 years].*

Despite these misconceptions, a minority of participants had undergone cervical cancer screening, and they reported varied experiences: some found it painless and straightforward, while others described discomfort, embarrassment, and fear, and they reported invasive procedures that they underwent. Notably, many women believed that the cervix is removed during screening, a misconception and mix-up between screening and treatment through conization (a minor surgical procedure in which a cone-shaped piece of cervical tissue is removed to treat precancerous lesions) that deterred others from participating.

*“For the others they say, the cervix is usually removed and put on*

*plate or extracted and it is put aside. What they heard is what they*

*usually pick up so you try as much as you can to explain the whole procedure*

*on how it is done, they end saying you can go, me for one am not going*

*anywhere” [FGD Participant, aged 46-65 years].*

### Barriers to cervical cancer screening

#### Cultural and social barriers.

Numerous barriers were reported and were categorized into sociocultural, informational, structural, and psychological factors. Culturally, participants across multiple FGDs held beliefs that cervical cancer was caused by witchcraft or was incurable, discouraging them from seeking medical interventions. Others believed that circumcision among Muslim husbands protected women, which reduced perceived susceptibility.

*“The other thing, a woman could say after all my husband is a Muslem since they are circumcised and hence, they are never dirty after all where will he have gotten the cancer that eats up the cervix, so because of that belief they would choose to stick to that instead” [FGD Participant, aged 25-35 years]*

Embarrassment and shyness also played a substantial role, especially among older women who expressed discomfort with exposing themselves to young or male health workers:

*“I can’t open up like that in front of someone of my daughter’s age” [FGD Participant aged 46-65 years]*.*“For me what I think that stops people from going for screening, for example I have seen a scenario where women run away from the queue at the health facility this is because some of these facilities use student doctors. For instance a women who is about 50 years cannot allow a young doctor examine them because they consider them their children thus leads up a situation where some leave the queue. Another thing is women are afraid of men examining them especially with the older years”[FGD Participant, aged 46-65 years]*

Several informants also reported barriers related to culture. Some women find it culturally not fit to be checked underneath, thus they find it as a burden, and this significantly influenced women’s decision to seek cervical cancer screening services, as one key informant reported;

*“And in my experience, I find that women do not want to get screened for cervical*

*cancer because their cultures do not allow them to insert things in their*

*underneath. Some women say they better go to traditional healers who*

*just give them medicines without opening their legs widely, and they*

*have the right medicines for their their problems, and they get cured*

*quickly”[KII_003]*

Similarly, social rumors and lack of confidentiality discouraged participation, and some think it can lead to death once screened. Participants reported undignified screening procedures, including lack of privacy and inappropriate comments from healthcare workers, were common deterrents.

*“Some women get scared to go for cervical cancer screening especially when they get reports from other people that have been screened. There is a case of a woman who went for the screening but after the test she became even worse until she died. So when someone hears such they decide not to go for the test so people have different perceptions but they are mainly based on what they hear from those that go for the test. Others wait when they get so ill”[FGD Participant, aged 46-65 years]*

#### Gender dynamics and power relations.

A recurring theme was the influence of male partners on women’s healthcare decisions. Many participants reported that their husbands either discouraged or outright prohibited them from attending screening services, citing jealousy or suspicion of infidelity.

*“If you tell him you want to screen, he asks, ‘Where did you get*

*the disease?”[FGD Participant aged 46-65 years].*

Several women described how patriarchal norms within their households, often led them to avoid the service altogether to maintain domestic peace.

*“… I for one think some women are being stopped to go for screening for cervical cancer by their husbands, some men tend to be tough on the women and they condemn them for leaving home to go take part in screening services I would think it is an obstacle for some women”[FGD Participant aged 36-45 years].*

This was also reported among the key informants that male partner disapproval significantly influenced women’s decision to seek cervical cancer screening services:

*“Some men refuse to allow their wives to go for cervical cancer screening, especially those who never went to school. They think that if she’s found with the cancer, it means he’s cheating or he is having another woman”[KII_001].*

Additionally, vulnerable women experiencing domestic violence or economic dependence were unable to make independent decisions regarding their health. Others were limited by their roles at home, particularly mothers without childcare support.

*“They cannot leave their children or house chores to*

*go for screening”[KII_002].*

#### Health system-related barriers.

Participants across multiple FGDs described how systemic barriers such as long wait times, understaffing, lack of equipment, inconsistence in service delivery, and charging for the services discourage women from seeking screening services. Additionally, women in Uganda often face unexpected charges for cervical cancer screenings due to corruption, despite services being advertised as free of charge. This financial unpredictability discourages many from seeking essential screenings, especially those with limited resources. Several women reported being told to return later due to absent staff or being asked to purchase gloves or other supplies, despite services being advertised as free.

*“The health workers always advise us to go there that the services are free of charge but when you reach there, the healthcare providers tell you to pay some money. So a woman may come from here knowing that the service is free of charge [inaudible voices in the background] and she carries only her transport but upon reaching there, they are told by health workers that the free things ended”[FGD Participant, aged 36-45 years].*

Additionally, the presence of only one screening device in some health centers led to extended waiting periods, deterring women who were balancing other domestic or work responsibilities. Privacy concerns, especially during mass screening events where multiple women were examined in the same room, further discouraged attendance.

*“When we went for that checkup, it was like an emergency, the health workers were putting four women in a room so there was an instance where they told an old lady to put off her clothes and the health workers realized she did not have an underwear so one of them shouted “you don’t even have an underwear?” so she felt ashamed”[FGD Participant aged 46-65 years].*

In line with reports from the FGDs, some key informants acknowledged that the attitudes of some health care workers also discourage women from screening, as one provider explained;

*“Sometimes you find that women will be having the knowledge they hear from*

*the radios about the cancer of the cervix. However, when she comes for*

*screening and the nurses alone are abusing everyone having done the*

*simplest of the mistakes during screening. Women will get discouraged from getting*

*the the screening services from such people and facilities. So, improving*

*the health care provider attitude in fact, if you have a poor attitude among your*

*women you may discourage many”[KII_004].*

Several informants acknowledged that the screening procedure is perceived as uncomfortable and invasive, often deterring women from participation. One provider explained,

*“It is very uncomfortable for women. You expose the most private parts, and this makes them uneasy… The position we put them in and ‘okwegaala’ [opening their legs widely] makes them very uncomfortable”[KII_003].*

Frequent stockouts of essential supplies such as acetic acid, insufficient staffing, and inadequate space for private examinations at healthcare facilities were cited as major issues hindering screening of women for cervical cancer.

*“We get people after health education and mobilizing, but then the materials to be used during screening such as the Acetic acid gets finished at the health facility. Due to this, even the nurse becomes demoralized”*. *You find that on such days, women that are screened are few, and it is not easy to get them again once they go back to their homes[KII_001].*

#### Knowledge gaps and misinformation.

A recurring view frequently cited was the inadequate health education and persistent misinformation as significant obstacles. Some women were unsure of the symptoms of cervical cancer, leading them to believe that the absence of pain itself meant absence of disease.

*“If I’m not feeling sick, why should I be screened?”*

*[FGD Participant aged 36-45 years].*

Health workers were criticized for not explaining the screening process or the disease in detail, contributing to knowledge gaps.

*“We the women are not so much aware about cervical cancer and therefore, we are not aware of its signs and symptoms [all participants repeat the same word] “we do not know them”. Such as, when I have this, it’s a sign that I could be having cervical cancer. So it requires these health officers to continuously sensitize us because when we visit the health centers they just keep on advising us to do cervical cancer screening but they don’t tell us”[FGD Participant aged 36-45 years].*

Myths around cervical cancer screening being painful or dangerous such as the belief that the procedure damages the cervix or involves unnecessary exposure were also prevalent. These misconceptions were often perpetuated by peers and, in some cases, even healthcare providers who failed to clarify doubts. Some women were reported to avoid screening for fear of pain or potential physical harm, while others feared being diagnosed with cancer itself.

*“There is a myth that when you’re screened for cancer of the cervix,*

*you will be found with it. So many people avoid it”[KII_004].*

Key informants noted that many women were unaware of the benefits of early detection and associated screening. As one stated,

*“Some women think the government wants to kill them like*

*that. They say things that are weird… they think it’s something*

*bad disguised as good”[KII_001].*

Additionally, the HPV vaccine is misunderstood and feared, with some believing it causes infertility or cancer:

*“They say that vaccinating children with the injection will*

*prevent them from giving birth”[KII_002].*

#### Motivators and facilitators to cervical cancer Screening.

Despite the barriers, several enabling factors were reported. Community sensitization and health education sessions especially those conducted by Village Health Teams (VHTs) and local health workers emerged as powerful motivators. Some participants appreciated being educated during routine visits or community outreach events, which made them more willing to engage in screening.

*“When health workers talk to us, we get the courage to screen”*

*[FGD Participant aged 46-65 years].*

Positive interpersonal interactions with healthcare workers also influenced uptake. Women described that

*“If the health worker is kind, we open up and get screened”*

*[FGD Participant aged 46-65 years].*

Several key informants also suggested that there is need to increase capacity of health care workers at the facilities such that they are skilled in performing cervical cancer screening procedures, One key informant mentioned,

*“I would like to suggest that the people in higher authority put in more*

*efforts in training other nurses at the facility such that they all learn this procedure*

*because the experience they get from training schools is too little….*

*Secondly, if they train a few of the nurses and are the only ones knowing how to do this, they become overwhelmed, and get tired easily, which leads to the poor attitude. And when the woman gets to the nurse when the nurse is already exhausted, they will not come back again for screening or they also discourage others from coming”[KII_001].*

For several participants, peer influence encouraged screening. Seeing their friends undergo screening without complications or witnessing someone recover from cervical cancer after early detection, reassured many women that the disease was manageable if discovered early. Participants mentioned that women can advocate for cervical cancer screening by employing a strategy of sharing real-life experiences and encouraging others to seek the service. Additionally, personal testimonies from those diagnosed and treated can motivate more women to get screened.

*“We can also try to show other fellow women how best to seek for the service and encourage them to seek the service bygiving live examples to them such that incase one has been screened and found with it they can say they were screened and also started their treatment; this would force others to also get to know where they stand in this case”[FGD Participant aged 36-45 years].*

Conversely, witnessing death from cervical cancer strongly influenced others to seek screening, particularly when the deceased was a known peer or community member

*“Doctor, what influenced me the most to go for cervical cancer screening was after I saw someone who died of it. That is when I decided to also go for the screening and see where I stand”[FGD Participant aged 25-35 years].*

*“What motivated [me] to be screened was the death of a friend. She was operated on and even they removed her cervix. So I make sure every August I go for screening”[FGD Participant aged 46-65 years].*

Several key informants noted that integration of screening into existing healthcare services such as HIV care and family planning as an important strategy to increase uptake of cervical cancer screening. Additionally, community-based education, drama, radio campaigns, and testimonies from cervical cancer survivors were also cited as effective strategies, as one of the key informant mentioned,

*“When survivors come back and talk, they recruit many other*

*women to test”[KII_003].*

Accessibility of services also played a role. The presence of nearby health centers and the possibility of walking or using low-cost transport (boda bodas) reduced logistical burdens. Some women even opted to pay for private screening services to avoid long queues at government facilities, emphasizing the importance of service quality and efficiency. Supportive spouses were another key motivator. While many women lacked support from their husbands, those who did reported that encouragement from their partners made them more proactive about their health. While awareness and service availability have improved over the years, key informants emphasized the urgent need to scale up targeted education, community outreach, and health system support to dispel myths, reduce stigma, and ensure sustainable access to quality screening services.

#### Suggested strategies for improving demand and uptake of cervical cancer screening services.

Participants offered numerous suggestions, such as community-based sensitization: using megaphones, radio, and television to broadcast accurate information. Providing pre- and post-counseling services to reduce anxiety, misinformation, and increase client confidence. Mandatory screening, including cervical cancer screening in antenatal care, family planning, and employment processes, was also key to improving participation in screening. Peer-led advocacy: Encouraging women who have been screened to share positive experiences. Health system reforms: Increasing staffing, ensuring the availability of screening equipment, and maintaining patient confidentiality. As one participant summarized.

*“Let them educate us well and handle us kindly. If we know the truth and are*

*treated well, we shall go for screening”[FGD Participant aged 25-35 years].*

The key informants also reported that while the screening services can not be extended to individual homes, they have introduced outreach programs bring the services closer to the communities, in order to improve access to screening services. One provider mentioned,

*“We now started to conduct community outreaches. In this, we bring*

*the services closer to where the women stay, although we cannot take the services*

*to their individual homes. For one who would want to access the service we usually gather*

*in the village-at a local council area, find a place where we can set up a community hub*

*and do the screening from there. So whoever wants the service, they can access the service*

*there. However, we have also noted that a few of the eligible women come for the screening services”[KII_002].*

## Discussion

This study offers critical insights into the socio-cultural, systemic, and interpersonal factors that influence cervical cancer screening behaviors among women in Wakiso District, Uganda. Through a qualitative exploration of focus group discussions and key informant interviews, the study revealed a complex web of knowledge gaps, fear, stigma, and health system challenges that collectively hinder uptake of screening services. These findings resonate not only with national studies in Uganda but also with broader evidence from Sub-Saharan Africa (SSA) and global contexts.

The persistence of misconceptions linking cervical cancer to family planning methods, poor hygiene, and witchcraft highlights persistent deficiencies in community health literacy and education. Similar misconceptions have been documented among women in Uganda [21,22], Kenya [23], Tanzania [24], Zimbabwe [25], and Nigeria [26], suggesting that misinformation remains a widespread barrier for cervical cancer screening across SSA. These misconceptions may reduce perceived benefits of screening and increase fear of participation. The Health Belief Model suggests that inaccurate perceptions regarding disease causation and susceptibility can substantially influence preventive health-seeking behavior. Beyond simply reflecting inadequate knowledge, these misconceptions appear to function as socially shared explanatory models through which women interpret cervical cancer risk within their everyday experiences. Rather than relying on biomedical explanations, participants often drew upon community narratives, cultural beliefs, and anecdotal experiences to explain disease causation. Such beliefs may create a false sense of protection for some women while increasing fatalism among others, thereby reducing both perceived susceptibility and the perceived benefits of screening. These findings suggest that improving awareness alone may be insufficient unless educational interventions explicitly address entrenched community beliefs and provide culturally acceptable explanations that replace misinformation with trusted biomedical knowledge. Our findings therefore underscore the need for culturally tailored communication strategies that directly address prevalent myths while providing clear information about HPV infection as the primary cause of cervical cancer. A prior review [27] found that myths about cervical cancer being caused by contraception, poor hygiene, or witchcraft were common across the region. In this study, similar misconceptions persisted, suggesting a critical need for targeted health education. These misbeliefs are not only inaccurate but dangerous, as they deter women from engaging in preventive behaviors like screening. Fear and fatalism further compound these knowledge gaps. Many participants believed that a diagnosis of cervical cancer equated to a death sentence. Importantly, the fear described by participants in this study extended beyond concerns about physical pain. Many women associated screening with embarrassment, exposure of intimate body parts, loss of dignity, or even permanent reproductive harm. These perceptions indicate that decisions regarding cervical cancer screening are shaped not only by knowledge deficits but also by emotional and cultural meenings attached to women’s bodies and reproductive health. Negative stories circulating within communities appeared to reinforce these fears, illustrating how individual experiences become collective narratives that influence health-seeking behavior across entire communities. In South Africa, Moodley et al. (2021) reported that fear of positive results discouraged women from screening, even when services were available [28]. In Uganda, similar themes have emerged, with fear of pain, embarrassment, and stigma all serving as barriers to screening [10]. These beliefs often stem from a lack of visible survivors and limited public discourse on successful treatment stories.

This study’s findings highlight the strong influence of healthcare providers on cervical cancer screening services. In contexts where providers were respectful, communicative, and culturally sensitive, women expressed willingness to return and recommend screening services to their peers. Conversely, negative experiences such as perceived rudeness, lack of privacy, or absence of female providers discouraged participation. This aligns with global evidence indicating that the quality of patient-provider interaction significantly affects screening uptake [29]. Training providers in compassionate care and ensuring gender-sensitive service delivery are essential components of successful screening programs. Another consistent theme across global and SSA literature is the role of peer and community influence. This study found that women were more likely to seek screening after hearing positive testimonies from friends or community members. Community-based mobilisation, therefore, holds strong potential. The study’s findings are consistent with findings from another study conducted in Uganda among rural women, which demonstrated that engaging community health workers and local champions can significantly improve awareness and service uptake [30]. Additionally, studies in Zambia and Nigeria also demonstrated similar findings on the role of community health workers and local champions in increasing screening awareness and uptake [19,31]. Uganda’s use of Village Health Teams (VHTs) offers a promising platform for scaling up such efforts. Our findings further suggest that trusted community actors play an important role in translating health information into action. Women consistently described being more receptive to information delivered through familiar community members, healthcare workers, or peers who had previously undergone screening. This highlights that trust, rather than information alone, may be the critical mechanism driving behavior change. Community-based interventions that leverage existing social networks therefore have greater potential to overcome misinformation and encourage screening than one-off facility-based health education campaigns.

Gender dynamics also emerged as a powerful force shaping decision-making for cervical cancer screening. In patriarchal settings, women often require permission from their husbands to access health services. Some participants in this study reported spousal resistance, with screening perceived as unnecessary or suspicious. Our findings are consistent with those from a recent study among rural women in Busoga Uganda, which highlighted the critical role of dialogue among partners in influencing decisions regarding cervical cancer screening uptake among women [21]. This barrier has also been echoed across SSA, where male involvement in reproductive health is often minimal, and programs in Kenya and Tanzania have begun involving men through couple-based health education, with promising outcomes in improving uptake and reducing gender-related stigma [32]. These findings also illustrate that cervical cancer screening decisions are embedded within broader household power structures. Women’s autonomy to seek preventive healthcare was frequently influenced by their partner’s approval, reflecting persistent gender inequalities in health decision-making. Consequently, interventions targeting women alone may have limited effectiveness if they fail to address household dynamics. Promoting constructive male engagement should therefore be viewed not as replacing women’s autonomy, but as creating supportive environments in which women are able to make informed decisions regarding preventive health services.

Systemic barriers including supply shortages, understaffing, and informal charges further limit cervical cancer screening access. Although screening is officially free in Uganda’s public health system, hidden costs such as buying gloves or transport fees disproportionately affect low-income women. Similar issues have been noted in Ethiopia and Ghana, where out-of-pocket expenses were major deterrents to screening uptake [33,34]. Policymakers must ensure that funding mechanisms are in place to sustain service delivery and remove financial barriers to improve service update. Importantly, participants rarely viewed these barriers in isolation. Long waiting times, shortages of supplies, concerns about privacy, and unfriendly provider attitudes often interacted to create cumulative barriers that discouraged women from returning for future screening. This suggests that women’s experiences within the health system substantially influence community perceptions of screening services. Improving client experience, including respectful communication, confidentiality, and efficient service delivery may therefore be as important as expanding the availability of screening services themselves.

This study also highlighted the potential of integrating cervical cancer screening into other reproductive and maternal health services. Participants suggested that offering screening during antenatal care (ANC) visits or immunization campaigns would improve service uptake. Similar findings have been shown in other prior studies in Uganda which suggest that cervical cancer screening services would easily been taken up if provided at similar points with other health services at the healthcare facilities [35]. A recent study conducted in Eastern Uganda among women at a health facility also highlighted the need to integrate cervical cancer screening services into existing community outreach programs such as immunization and family planning to improve the uptake of screening services [36]. Similar evidence from other African countries (Rwanda and Malawi) supports this integration approach, which has been shown to increase coverage without overwhelming existing systems [37]. In Uganda, where ANC attendance is relatively high, leveraging these touchpoints could significantly improve screening rates. From a policy perspective, the study supports the need for a multi-pronged, context-specific strategy. Educational campaigns must be localized, involve trusted community figures, and incorporate culturally relevant content. Health systems must prioritize respectful, confidential, and accessible service environments. Community engagement, particularly through testimonies from survivors and outreach by VHTs, is critical to reducing stigma and building trust. Integrating cervical cancer screening into existing maternal and reproductive health services may also help normalize screening as a routine component of women’s healthcare rather than an exceptional procedure sought only when symptoms develop. Such integration has the potential to reduce stigma, capitalize on existing healthcare contacts, and improve early detection while minimizing additional costs for both women and the health system. These findings therefore support a shift from opportunistic screening toward a more integrated and person-centred model of cervical cancer prevention.

Importantly, this research provides evidence for rethinking national cervical cancer prevention strategies. Uganda’s National Strategic Plan (2020–2024) outlines goals for HPV vaccination, screening, and treatment. However, achieving these goals will require overcoming entrenched social and systemic obstacles, as documented in this study. Greater investment in community education, healthcare provider training, and mobile outreach services is essential. Collectively, these findings demonstrate that increasing screening uptake requires interventions operating simultaneously at individual, community, and health-system levels. Addressing only one dimension for example, improving awareness without strengthening services quality is unlikely to produce sustained improvements in screening uptake. Instead, comprehensive interventions that combine culturally responsive health education, community engagement, male partner involvement, and health system strengthening are likely to have the greatest impact on achieving Uganda’s cervical cancer elimination targets.

The study’s qualitative design enabled a rich, in-depth understanding of the factors influencing screening behavior. However, it also has limitations. The findings are context-specific and may not fully reflect the diversity of experiences across Uganda. Moreover, the perspectives of men and healthcare policymakers were not thoroughly explored. Future research could build on these findings through larger mixed-methods studies or intervention trials to test community-driven strategies.

## Conclusion

This study provides important qualitative evidence on the perceptions, experiences, and barriers influencing cervical cancer screening among women in Wakiso District, Uganda. Although awareness of cervical cancer exists, misconceptions regarding disease causation, fears related to screening procedures, gender power dynamics, and health-system challenges continue to hinder screening uptake. These findings demonstrate that improving screening coverage requires more than service availability alone, but rather community-based health education, male partner engagement, respectful and confidential service delivery, and integration of screening into routine reproductive health services are critical strategies for increasing participation. Addressing these barriers is essential for Uganda to achieve national cervical cancer prevention targets and contribute to the WHO’s goal of eliminating cervical cancer by 2030.

## Supporting information

S1 File S1 Text. (DOCX)

S2 File S2 Data. (DOCX)

S3 File S1 Data. (XLSX)
