# Supplementary material for: “Even with one Man, but with many women, you can get cervical cancer”: Qualitative exploration of women’s perceptions and experiences of cervical cancer screening in Uganda: A community-based study
Source: PLOS Glob Public Health. 2026 Sep 18;6(9):e0007256. doi: 10.1371/journal.pgph.0007256 (PMC13588353; doi:10.1371/journal.pgph.0007256)
Supplement: Supplementary file 1 [file pgph.0007256.s001.docx]

**FGD GUIDE For Cervical Cancer Study.**

**Project Title:** Cervical Cancer Study

**Assessing the Perceptions, Attitudes, Knowledge (PAK), and Barriers associated with Cervical Cancer Screening among Women aged 25-65 years in Wakiso district, Uganda**

**Principle Investigator:** Mr. Robert Bulamba

**Introductions:** Hello, my name is ______________________ I work with Africa Medical and Behavioral Sciences Organization. We want to assess the Perceptions, Attitudes, Knowledge (PAK), and Barriers associated with Cervical Cancer Screening among Women aged 25-65 years in Wakiso district, Uganda. In reference to the consent form which you have read/has been read to you, and already signed, do you have any questions for me before we begin?

**Cervical Cancer Screening among Women above 24 years, Wakiso district: Assessing Knowledge, Perception and Uptake, in a population based cohort.**

**Questions;**

| **A: Knowledge on Cervical Cancer Screening** | |
| --- | --- |
| **No.** | **Question** |
| We are interested in hearing about health issues among people in your community, and particularly about Cervical cancer and its screening. | |
| 1 | What knowledge do you have about cervical cancer and ***(prob: for screening methods such as HPV, Pap smear etc...)***  Luganda: |
| 2 | What are the prevailing myths and misconceptions surrounding cervical cancer and its screening among women in your community?  Luganda: |
| 3 | What role do healthcare providers play in promoting cervical cancer screening among women in your community?  Luganda: |
| 4 | How does knowledge about cervical cancer signs and symptoms, and risk factors relate with women's likelihood to seek screening in your community?  Luganda: |
| 5 | In your opinion, what are the factors that influence women’s participation in cervical cancer screening?  Luganda: |
| 6 | How do personal experiences influence women's decisions regarding cervical cancer screening in your community?  Luganda: |
| 7 | What interventions can impact women's knowledge, attitudes, and practices regarding cervical cancer screening in your community?  Luganda: |
| 10 | What are the perceptions and experiences of women who have undergone cervical cancer screening in your community?  Luganda: |
| 11 | What cultural beliefs and practices impact the women’s participation in cervical cancer screening among women in your community?  Luganda: |
| 12  13 | How does access to healthcare facilities affect the participation of women in cervical cancer screening programs in your community?  Luganda:  b)How can these be improved?  Luganda: |
| **B: Factors influencing cervical cancer screening.** | |
| Now, let us discuss about issues that may influence cervical cancer screening in your community | |
| **No.** | **Question** |
| 14 | How does socio-economic status influence awareness and knowledge of cervical cancer screening among women? ***(Prob: for where they first learnt about screening)***  Luganda: |
| 15 | What factors are responsible for the low participation of women in Cervical Cancer screening among women in your community? ***(Prob: for partner support ie financial, social support etc...)***  Luganda: |
| 16 | What can be done to increase access to cervical cancer screening? ***(Prob: for community-based screening programs)***  Luganda: |
| 17 | How does awareness about cervical cancer screening methods affect women’s participation in cervical cancer screening in your community?  Luganda: |
| 18 | How do healthcare providers influence women's willingness to participate in cervical cancer screening programs in your community? ***(prob: referals, counselling etc.....)***  Luganda: |
| 19 | What barriers deter women from seeking cervical cancer screening services in your community? ***(Prob: for distance, transport, fear healthcare attitude etc..)***  Luganda: |
| 20 | How can these barriers be addressed to improve women’s participation in cervical cancer screening programs/services?  Luganda: |
| **C: Attitudes towards cervical cancer screening** | |
| **No.** | **Question** |
| 21 | In your opinion, what are your perceptions of the effectiveness and reliability of cervical cancer screening methods currently being used?  Luganda: |
| 22 | How does religious beliefs influence women’s perception and acceptance of cervical cancer screening among women?  Luganda: |
| 23 | What are the perceptions and attitudes of women towards cervical cancer screening methods in your community?  Luganda |
| 24 | b) How does the above influence women participation in cervical cancer screening in your community?  Luganda |
| 25 | What family dynamics influence women's decisions to undergo cervical cancer screening in your community?  Luganda: |

**KII GUIDE – For Cervical Cancer Study.**

**Project Title:** Cervical Cancer Study

**Assessing the Perceptions, Attitudes, Knowledge (PAK), and Barriers associated with Cervical Cancer Screening among Women aged 25-65 years in Wakiso district, Uganda**

**Principle Investigator:** Mr. Robert Bulamba

**Introductions:** Hello, my name is ______________________ I work with Africa Medical and Behavioral Sciences Organization. We want to assess the Perceptions, Attitudes, Knowledge (PAK), and Barriers associated with Cervical Cancer Screening among Women aged 25-65 years in Wakiso district, Uganda. In reference to the consent form which you have read/has been read to you, and already signed, do you have any questions for me before we begin?

**Cervical Cancer Screening among Women above 24 years, Wakiso district: Assessing Knowledge, Perception and Uptake, in a population based cohort.**

**Questions;**

| **A: Knowledge about Cervical Cancer Screening** | |
| --- | --- |
| **No.** | **Question** |
| **1** | Tell me about your experience with cervical cancer screening? ***(Probe for any methods ever used)***  ***Luganda:*** |
| **2** | What factors do you believe influence women to participate in cervical cancer screening programs / services?  Luganda: |
| **3** | What are the limiting factors to women to their participation in cervical cancer screening services /programs?  Luganda: |
| **4** | What specific age groups or life stages women are more or less likely to prioritize cervical cancer screening?  Luganda: |
| **4** | What barriers or challenges have you encountered in promoting cervical cancer screening?  Luganda: |
| **5** | How do you encourage women to participate cervical cancer screening services?  Luganda: |
| **6** | What improvements or changes would you suggest to health officials to improve cervical cancer screening services/ programs that are currently being provided?  Luganda: |
| **7** | How can cervical cancer screening services be better integrated into other existing point of care services at healthcare facilities?  Luganda: |
| **8** | How do you perceive the importance of early detection through cervical cancer screening?  Luganda: |
| **9** | What are some of the myths and misconceptions about cervical cancer screening have you frequently encountered?  Luganda: |
| **10** | a.How do you address them?  Luganda: |
| **11** | What strategies do you employ to ensure adequate follow-up and management of patients with abnormal cervical cancer screening results?  Luganda: |
| **B: Attitude towards Cervical Cancer Screening** | |
| **13** | How do cultural beliefs and practices affect attitudes towards cervical cancer screening in your community?  Luganda: |
| **14** | What strategies have been effective in promoting cervical cancer screening awareness and participation in your community?  Luganda: |
| **15** | How would you describe the general awareness level among the community regarding cervical cancer screening methods?  Luganda: |
|  | How do women's attitudes towards preventive healthcare in general influence their likelihood of obtaining cervical cancer screening?  Luganda: |
| **C: Factors influencing Cervical cancer screening** | |
| **16** | How do socioeconomic factors, (such as income level and education,) impact women's likelihood of undergoing cervical cancer screening?  Luganda: |
| **17** | Discuss how religious beliefs may affect women's attitudes towards cervical cancer screening?  Luganda: |
| **18** | a.How do you address these beliefs in your programs?  Luganda: |
| **19** | What role do the awareness campaigns and educational initiatives play in influencing women to seek cervical cancer screening?  Luganda: |
| **20** | How do women's perceptions of their own health and vulnerability to cervical cancer impact their decision to undergo screening?  Luganda: |
| **21** | How do family and social networks influence women's participation in cervical cancer screening?  Luganda: |
| **22** | What role does fear of the screening process or fear of receiving a cancer diagnosis play in women's decision-making regarding cervical cancer screening?  Luganda: |
| **23** | How do women's past experiences with healthcare services, including cervical cancer screening, shape their willingness to participate in future screenings?  Luganda: |
| **25** | How do logistical factors such as transportation, childcare, and time constraints impact women's ability to access cervical cancer screening services?  Luganda: |
| **26** | a.What strategies are in place to address these barriers?  Luganda: |
|  |  |
