# Supplementary material for: “Even with one Man, but with many women, you can get cervical cancer”: Qualitative exploration of women’s perceptions and experiences of cervical cancer screening in Uganda: A community-based study
Source: PLOS Glob Public Health. 2026 Sep 18;6(9):e0007256. doi: 10.1371/journal.pgph.0007256 (PMC13588353; doi:10.1371/journal.pgph.0007256)
Supplement: Supplementary file 2 [file pgph.0007256.s002.docx]

**SECTION A**

**General results from Women**

**Study Title: Assessing the Perceptions, Attitudes, Knowledge (PAK), and Barriers associated with Cervical Cancer Screening among Women aged 25-65 years in Wakiso district, Uganda**

**A-KNOWLEDGE ABOUT CERVICAL CANCER SCREENING**

# 1.0. **Experience with Cervical Cancer screening**

Participant discussed their experiences with cervical cancer by highlighting their knowledge about the perceived causes of cervical cancer. The participants discussed a number of factors they attributed to cervical cancer among the women. Among them, they noted poor personal hygiene among women, having sexual intercourse with multiple sexual partners, having sex with uncircumcised men, having sex before healing after birth and having sex during menstruation period. Additionally, participants perceived that some contraceptive methods such as IUD can cause cervical cancer.

From their experiences, some participants perceived that a wife can still get cervical cancer if her husband has unprotected intercourse with another partner who carries the virus, even if she has only been with him.

“Doctor me I think even when you have not engaged with different men but still have your husband at home but he still sexually has intercourse with madam XX without using protection I think he can be able to transmit that disease. All that I know if at all one sexual partner has the cancer of the cervix virus by all means me the wife of home can as well get the cancer of the cervix.” **[FGD_001]**

The participant believed that cervical cancer is caused by men and understood that, when contracted, it does not manifest immediately.

“What I know about cervical cancer [participant clears her voice] is that the virus is found on the cervix and it is in most cases spread by our by men and when it has entered you, you can’t feel it because it doesn’t immediately show the signs. The little I know is that, when that virus is quickly attended to there is a procedure they undertake to treat it before it worsens but only if you attend to it quickly. This virus is commonly spread by the men to the women at least I know that one better.” **[FGD_002]**

“I mean being careful about the sexual partners because some men can transmit different diseases [meaning sexually transmitted diseases] to women resulting into cervical cancer. So I think it is important to always know our status by carrying out cervical cancer screening.” **[FGD_003]**

“Having sexual intercourse with uncircumcised man also causes cervical cancer. It is claimed that when uncircumcised man goes into sexual intercourse with an infected woman, he can easily acquire the HPV and carry it under his foreskin to another woman without being affected. That is what we were taught [laughter].” **[FGD_003]**

“Young girls who involve themselves in sexual activities with older men, they can easily get cancer of the cervix. Some girls begin using family planning while still young up to the university level and they keep on switching from one method to another. These family planning methods usually cause cysts or swellings within the uterus and they end up getting cancer of the cervix.” **[FGD_004]**

“Personally, I think if a woman engages with multiple sexual partners and they also don’t use protective things like the condoms, it is very easy for them to get cancer of the cervix. Another issue is when they use these condoms wrongly it can also cause cancer.” **[FGD_004]**

“What I know about cervical cancer is that, it is spread by men from one woman to another through having sexual intercourse.” **[FGD_005]**

“My submission is similar to that lady, that cervical cancer is spread by men and it is very common among the married women in that sometimes your husband may commit adultery with a woman who has it and as he comes back and have sexual intercourse with you, he passes it onto you. That is what I know about it.” **[FGD_005_02]**

“Me I think cervical cancer comes when a woman is in her menstrual periods then she engages in sexual activities with a man, that blood can clot and cause cancer of the cervix.” **[FGD_004]**

Engaging in sex during menstruation may delay blood flow, potentially leading to cervical cancer. Additionally, stopping sexual activity while still physically active could cause fat accumulation and clots, which may contribute to the disease.

“What I would think about as something that could lead to cervical cancer in women could be that while one is in their menstrual periods and still engage in sexual activity while you are still in that period, the blood might be delayed to be pushed out that alone could lead to cervical cancer. The other thing women, in case a woman quits engaging in sexual activity while still active this can lead to cervical cancer how, this leads to excessive fat in the body form a clot this could lead to cervical cancer since it is not being pushed out that is what I would think about.” **[FGD_001]**

Some participants attributed the cause of cervical cancer to the practice of restricting blood flow during menstruation.

“For me I think, for the women that get in to their menstruation period then they use medicines that stops the blood flowing yet the blood is meant to flow, each time you prevent the blood from flowing out the blood clots and hence leading to cancer of the cervix.” **[FGD_001]**

Participants noted that resuming sexual activity before fully healing after childbirth may increase the risk of cervical cancer.

“Some women after giving birth they do not give themselves time to heal completely. Before the uterus settles into its place, they are already engaging in sex and it makes it worse if they are not having sex with one man, so this affects the uterine muscles and it weakens then that puts her at risk of getting cancer.” **[FGD_004]**

Some participants perceived that planning methods are dangerous to their body so they can expose a woman to risks of acquiring cervical cancer. They added that, some of these methods stay in the body for so long and they make a woman prone to acquiring the virus.

“I think in most cases this disease [referring to cervical cancer] is caused by sexual intercourse and the use of various family planning methods. So if the use of such methods is reduced and also women visit the healthcare facilities in time, cervical cancer can be prevented.” **[FGD_003]**

“Some people say that there is a specific type or method of family planning called IUD [Intra-Uterine Device], that it is the one that causes cancer of the cervix. When someone stays with it for a long time in the body by the time they remove it someone could have gotten cancer.” **[FGD_004]**

“I heard that according to family planning methods we use especially IUD (Intrauterine Device) causes cervical cancer. They even gave an example that if a metal can rust when placed on a mere substance, how dangerous could it be in the human body?” **[FGD_006]**

“This family planning of pills also puts women at risk of cervical cancer. Some women use it for a period of three to five years so the place where these pills are deposited onto the uterus develop cysts which consequently affects the entrance of the uterus consequently develops into cervical cancer.” **[FGD_004]**

“What I know about that disease is that family planning methods have contributed a lot to the spread of cervical cancer, all those family planning methods including condoms. I once used the family planning pills but I could reach a moment… “When you don’t want to get sick, do not swallow the red pills.” I also used not to swallow them. We have just discovered after sometime that they are very dangerous because they stop the blood which is supposed to come out from coming out. We are just realizing it that they too have contributed a lot to the spread of cervical cancer.” **[FGD_002]**

A number of participants perceived that using public toilets without water for hygiene may expose women to infections. This was as indicated in the following quotations.

Me what I think when we use these public toilets without having small jerry cans of water for us the women for use while there, when we use the toilets those tinny viruses in there can get into pour private parts and if one does not take the trouble to treat themselves one can be able to get cervical cancer. **[FGD_001]**

Poor hygiene in the toilets. I can talk about squatting in the dirty washroom where one has urinated. You need to go there when it is clean so as to keep good hygiene just as this lady said, it is indeed very true. Personal hygiene is crucial especially cleaning the nickers. Even adulterous men can spread the virus for that disease. **[FGD_002]**

About this cancer, I can talk about the poor hygiene among the women whereby someone might wear a nicker for a full year and still you carry it to the next year. It would be better for a woman to dress like three knickers a day and also taking time to maintain good hygiene. **[FGD_002]**

I think cervical cancer is caused by poor hygiene for example, sometimes we [referring to ladies] add artificial nails onto our fingers which can accidently damage the cervix while bathing leading to cervical cancer.” **FGD_003]**

Some participants had undergone cervical cancer screening and possessed sufficient knowledge of the screening process. While some reported no issues with the procedure, others mentioned concerns about privacy, pain, and discomfort during the screening. It is worth noting that a few participants did not understand the screening procedures so, they had scanty information. This is expressed in following quotation.

For me I have ever screened for cervical cancer but I did not understand the process, they place a material very cold around the cervix. After the screening they brought for me results claiming I do not have it has taken me around 7 years ever since that time I have not yet screened again. **[FGD_001]**

I also had fear to participate in cervical cancer screening thinking that the process is painful or involves opening a person widely [laughter] but I didn’t experience any pain during the screening process. So I argue the fellow women participate in cervical cancer screening because it has no negative effects. **[FGD_003]**

All I know about screening for cervical cancer, me personally I have screened about 8 times or even more they usually give me a period of about 3 years until the next screening, they usually provide you with a card if at all women adhere to this that for a period of time given to them. I have been going for this from the time I was about 25 years and I do not have any issues with it actually my screening time is still valid because they had just screened me last year. **[FGD_001].**

While I was being screened in the machine, for me I did not get any issues, for those that say the cervix is usually removed that is not true the machine is brought closer to your private part and it enlarges a bit so that they can clearly see the cervix, all this is cast on a television sort of machine I was able to see all this and they did ask whether I was able to see my cervix and they concluded it had no issue and I did not get any issue. **[FGD_001]**

Me I have ever tested, the Kabaka of Buganda was coming to the county grounds and there was that service of routine cervical cancer screening so I went and I was screened but according to way we were screened I wonder if there is another better than that! Imagine it is like someone is giving birth, they take off all your clothes, then they begin to check from the chest area of the body, then the insert a machine into the vaginal canal while your legs are spread apart. But I would like to know if there are other methods better or other than the one they used to screen us. **[FGD_004]**

… A health worker puts on gloves then there is a device shaped like a needle though bigger than a needle that they insert in the birth canal then they get out some fluids but when you are in that position it appears like you are giving birth to a child. After a short while they write to you and inform you whether you are sick or not. **[FGD_004]**

You just visit a health center because cervix screening is something easy to do. It may appear complex if you haven’t experienced it. They use something that is just like this pen and they first make you lay on a bed however, it is not that bad to the extent that number one explained that they insert a machine in you on this side and the other. You just squat a bit, and the health provider brings something that is like a pen and he passes it like this [participant demonstrates rotation of the machine]. It is something small that you can’t even realize when they pass it and after they tell you to… it is something easy though when we are this side and we haven’t experienced it… when someone comes and narrates it to you just as number four said that they insert a hand in you and they remove the cervix, you cannot get courage to go for screening. It requires when you go there by yourself because even the minutes we have so far spent here are much if you had gone to screen for cervical cancer. They can approximately screen seven women in the minutes we have so far spent here if one had gone just for screening because it takes around three minutes. **[FGD_002]**

There is a method used which has scared most women; it involves the health worker telling you to undress yourself and sleep on the bed, you put the legs on either sides and she inserts a machine so that it opens, after she brings a pair of scissors that contains some cotton and he does like this [participant rotates her hand]so that he is able to do what… because as they are screening, you may feel like something is itching you and as she is passing it on the cervix to see whether it contains the viruses. They do so using the machine that I have told you about that they pass there like this so that it widely opens like this [participant widens her arms to demonstrate widening]. So after you opening widely and him drawing their some light to see clearly, he comes and starts to clean on the cervix to see whether it has the viruses and he puts them somewhere and he takes them. There is also another method that I witnessed; there is something that is in a pen like structure which appears to be having some cotton. She and insert inside there and after he returns it back and cover it… this is the easiest method. **[FGD_002]**

… all of them were performed on me because I had a child at Delilah... and it was a must to be and in case you were not interested in cervical cancer screening you were allowed to move out. At first I was not interested and it pressed me harder because I didn’t go there when prepared for the screening but I dedicated to it and all the procedures she has spoken were done on me. They used that pen like machine which she has said that has… there is very little pain you feel and in just a minute you be done. There is a day I wanted to get screened in Kawaala [participant refers to Kawaala health center 3] but they said that, since am beyond 50 years of age, if am interested in the screening, I should go to Kawempe [participant refers to Kawempe regional referral hospital] though I have not yet went there. I have so far screened for cervical cancer at Delilah two times and there is no complication with it. **[FGD_002]**

That machine just opens the front opening so that they can easily see the cervix. They have a plastic machine which is has a sticklike structure and it has bristles like those of a toothbrush, though these ones are surrounded on the machine. So when they insert that machine which is called vaginal… it’s a vaginal what! It is just a machine that opens and it has a name which we call it, though I have forgot it, so when it opens it like this [participant demonstrates the opening of the vagina by widening arms] then the health worker inserts the other stick like machine and passes it on the cervix like this [participant demonstrates like rotating the stick like machine on the cervix] as he is rotating it [participant claps her hands after speaking]. So when he removes it, it’s the one that he stores in its stuff which is just as like this bottle, he closes and tighten it well. That, it is what they take in the laboratory, the first thing they observe in it is that, does it contain blood or not? And if it has blood, you are more likely to be having that cancer. So, when they take it in the laboratory, they screen it. Even to the other procedure of doing like this [participants rolls her hand], they just remove that fluid to test the presence of cancer cells and after they give you the results. They may. **[FGD_005]**

To where I went, I slept on a bed and put one leg on one side and another one to the either side,[participants laugh] they have a machine which they insert in you and they rotate it so that it enlarges and thereafter they use a torch to draw light there and they [participant laughs] start doing something there inside… it could be cotton that they insert there using a machine and start doing like this [participant demonstrates how they operate a machine] but the machine they use to widen you there is a way thy operate it to widen you better and when they are done... When they are done…, to me they immediately told me to go that I didn’t have it. They didn’t wait for hours or to take me to the laboratory. I have so far been screened two times still from that same place. **[FGD_005]**

For my sake, they used a peg-shaped instrument [referring to speculum] which was placed in the vagina [to allow visualization of the cervix] and a cotton swab [referring to cervical sampling swab] that was used to collect cell samples from the cervix to examine for the presence of HPV. **[FGD_003]**

I have ever been screened from XX health facility but the screening process involved bringing the cervix closer to collect some fluids that is used to detect cervical cancer. **[FGD_003]**

I was also screened from XX health facility but I didn’t understand the instrument that used however they placed some cold substances into the vagina which but I didn’t feel any pain during the process. **[FGD_003]**

# 1.1. **Factors influencing participation**

*Health education and sensitization session.* The majority of participants noted that health education and ongoing sensitization sessions about cervical cancer screening are key factors influencing women's participation in screening. They discussed how these sessions raise awareness and motivate women to undergo screening. This is further illustrated in the following quotation

“The village health workers have taken the trouble to sensitize us, each time you meet the village health worker they usually share with you all that concerning cervical cancer but we the women do not consider that important they talk about it and leave it at that.” **[FGD_001]**

“I think one of the motivators is sensitization. For example, I was motivated to take part in screening after several sensitizations by the health workers. So we get motivated to participate in the program whenever the health workers reach out to us in our communities.” **[FGD_003]**

“Just like my colleague has said…. we used to have such services in this community sometime back. They used to come and teach us and we would even register then they would take us to the health facility early in the morning and everyone would come back checked, assessed and given treatment and medication if they were found ill or with a certain disease.” **[FGD_004]**

“The health educative talks really help a lot especially to those who are more concerned about their lives, yes it is true that people are more attracted to something that gives them money but still if someone is more interested in a better health they will go for the test. Some of us who have aged we know what it means to go for these tests that we even pay for. People keep with illnesses simply because they are waiting for services where they are given money in return.” **[FGD_004]**

Healthcare providers play a pivotal role in encouraging women to undergo cervical cancer screening. By offering comprehensive health education, they emphasize the importance of early detection and understanding one's health status. This proactive approach not only raises awareness but also empowers women to take preventive measures. This is explained further in the following quotation,

“The health workers usually take care as long as you go to the health facility, in this the make you like the whole processes involved as they make you aware that even if you had gone for something else, you end up going for screening. They accord you the necessary time in the end the care that is at the section lures one to screen for cervical cancer.” **[FGD_001]**

“The health workers raise our hope and morale to participate in screening when they counsel us even after one tested positive and cancer hasn’t reached stage three, it becomes easy for the them to treat the disease or to spare the woman’s life by removing the cervix in order to enable her have a better future to her children. They also advise us to avoid such challenges by carrying out early cervical cancer screening. They also encourage women to go for cervical cancer screening every after a year.” **[FGD_006]**

“We always get health workers in our community and also the vehicles move around alerting us about the health camps. Even the VHTs educate people in their respective communities so we always get different health workers however some women make themselves too busy to get time for such programs.” **[FGD_003]**

“First of all when you go to the health facility, the health worker you find there is able to tell you about the screening of cervical cancer, in this one is able to be persuaded to go for the screening after them taking you through what it is about the you can be able to at least screen.” **[FGD_001]**

“… the services are always available because health workers normally reach out to our communities to mobilize women participate in the screening. Even if someone decides to access Kawaala health facility, she can easily do so because it is near but some just either have fear or don’t give the service a priority.” **[FGD_003].**

In most cases, the health service providers that come into our communities deliver such services on a voluntary basis but some people don’t want to participate in cervical cancer screening. **[FGD_003]**

“They also use televisions to sensitize people as well as encouraging them to participate in cervical cancer screening.” **[FGD_003]**

“One day as I was taking my child to XX health facility for immunization, I found the health workers encouraging a group of women to carry out cervical cancer screening. They told them that when they delay to carry out screening, their life span is reduced but when they are screened positive at an early stage, they immediately put you on medication.” **[FGD_003]**

The treatment a woman receives from health workers can significantly impact her willingness to return for cervical cancer screening. Unfair or poor treatment at facilities can discourage women from seeking services again. Additionally, the reception women receive from healthcare providers plays a crucial role in their willingness to undergo cervical cancer screening. Positive and welcoming attitudes encourage openness, while rude or dismissive behavior can deter women from seeking care or getting screened. This was illustrated in the following quotations,

“In most cases, the way a woman is treated by the health workers of different health facilities can kill that person’s morale to revisit such facilities for cervical cancer screening services. For example, you [referring to a woman] visit the health facilities like Kawaala, Kawempe or Nabweru and the health worker [takes you speed] meaning unfair treatment can also prevent women from participating in cervical cancer screening.” **[FGD_003]**

Healthcare providers are not bad people but each one has their own character traits some are rude others are welcoming so the reception that a woman gets from a healthcare provider determines their level of openness to the same. A positive reception will eventually lead to more women being screened in whichever illness that someone might be having. Some health workers use very bad language for instance she can tell you [“sijja kubera kugwe weeka yogera ekyakulese”] meaning [“I am not here to work on you alone, speak out why you are here”]. So you wish that may be you could have found another person. **[FGD_004]**

Participants reported that witnessing women die from cervical cancer could motivate them to get screened and better understand their own health status. This is noted in the following quotation.

“Doctor me what influenced me the most to go for cervical cancer screening was after I saw someone who died of it that is when I decided to also go for the screening and see where I stand.” **[FGD_001]**

What motivated to be screened was the death of a friend. She was operated on and even they removed her cervix. So I make sure every August I go for screening. **[FGD_003]**

Participants mentioned that seeing colleagues recover from cervical cancer after treatment reassures women that cancer is not an immediate death sentence. This instills confidence in them to go for screening. This was reported in the following quotations.

“For me to go and screen I noticed some lady that had been screened and diagnosed with cervical cancer and they are yet to die, “they claimed” but when they took her and put her on treatment and told her that whoever contracts cancer does not easily die and they were promised to be given treatment and they would heal, they took about 3 years while not so well but eventually they slowly started to get better that alone persuaded me to go for the screening.” **[FGD_001]**

“For me I draw my motivation from my other colleague who has just been screened and later found that she has cervical cancer but now she is on treatment so this am also motivated to go and screen for cervical cancer because it is a free service.” **[FGD_001]**

For me where I would be persuaded to go screen for cervical cancer would be reference from my other fellow woman who decided to go for the screening thus, I could be able to give in and also go. **[FGD_001]**

Participants highlighted that health facilities are conveniently located nearby, making them easily accessible. They emphasized that the minimal transport costs required to reach these facilities further encourage women to seek cervical cancer screening and other healthcare services. This accessibility reduces financial and logistical barriers, enabling more women to prioritize their health.

“We have health facilities that are close by to a point that with even one thousand shillings as transport you can get there, for example we have Kawaala health facility is close by however just as the ladies said sometimes you could go there and they have excuses such as we are already off duty but all in all the facilities are close by.” **[FGD_001]**

“The health facilities are closer because one is in Kawempe and the other one this side in Kawaala am not sure whether even in Nabweru they also screen. Are those places really far? You may even just walk there to go for screening. Previously people used to travel to Mulago but nowadays they go to Kawempe and Kawaala.” **[FGD_002]**

“The distance has no effect on women’s participation in cervical cancer screening because the health facilities within our community are in a walkable distance. When a person wants to reach the facility in time, it takes her only one thousand shillings to get a ‘boda boda’ [meaning a public motorcycle and then return on foot].” **[FGD_003]**

“From here to the healthcare facility is not far, someone can even walk there. It is someone’s willingness to go for the screening service but women are generally sluggish in seeking healthcare services.” **[FGD_004]**

The availability of cervical cancer screening services in private clinics encourages some women to undergo screening. This is particularly beneficial for women who prefer to avoid the long wait times at public health facilities and are willing to pay for quicker, more convenient services. This is as explained the quotation below.

“Doctor me what I want to supplement on is, amongst us here there women that work it is possible to earn some money one can choose to visit a hospital where you pay money with intention to go for screening than rather lagging back in government facilities with free services were you cannot get service as fast as possible the way you need it .It is rather best choosing to go to a facility even if it is for paying money so that you can ensure that you still catch up with the time one has to work please ladies we should not keep our selves at the back.” **[FGD_001]**

Some participants explained that supportive husbands who encourage their wives to go for cervical cancer screening play a crucial role in motivating them to prioritize their health.

“For our husbands, if a woman claims that they may need to go for cervical cancer screening you may not ask why but would rather motivate me more to go for the screening to see where you stand.” **[FGD_001]**

The way women are treated during screening services significantly impacts others' willingness to participate. Negative reports, such as uncomfortable or invasive experiences, can deter others from attending.

“I would say that the way how women are handled when they go for these services is very important. The reports that we get from those that have gone for the service contribute much to others participation. Usually, the reports that come from those that have tested are not good so others are discouraged to go. For instance, the reports that came from the camp last time which even members here have told us, they do not entice others to go for the test, four people in a room yet they undress you while others are watching, that is not right. Women should also be taught that when you come from the screening exercise please do not discourage others by giving them a bad report because it discourages them.” **[FGD_004]**

# 1.2. **Strategies of encouraging women to participate in cervical cancer screening**

Participants suggested that workshops and counseling can encourage women to attend cervical cancer screening. Additionally, they advised that welcoming healthcare environment and proper guidance are key to increasing participation.

They should set up workshops and they sensitize women and also interest them to attend cervical cancer screening… “it has no complications but if you refuse, you may get other complications”. Because as you can see, previously we used to go to Mulago for antenatal care services and they could first offer us some counseling on different things so that by the time you go, you are well acquainted with something and how it is done such as when a child is growing you can handle it in such and a such a way however, these days you may go to a health center like Kawaala and you tell them that I have come for screening and they say that please sit there and wait on your fellows to come or sometimes they tell you that the person who offers that service is not yet in place. They do not show any love which may make you decide to go back with your disease. We used to visit Mulago for the maternal services but they could first offer us some counseling. There could be some women whom am not aware whether they are still there but they could sensitize us about feeding, bathing among others and by the time you go out you have gained some knowledge about something but these days… **[FGD_002]**

They have to provide counseling to us so that they capture our minds and heart so that we put trust in them. **[FGD_002]**

Workshops with experienced counselors can raise awareness about cervical cancer and encourage cancer screening. Real-life survivor stories can help reduce fear and motivate a good number of women participate in screening.

When they organize a workshop and people gather… since the cervical cancer outbreak started, it cannot be reversed. Its causes are also very many which I do not think that they will go back soon because some are like required in life whereby you can’t forge life without them. If we could get a workshop with good counselors who are experienced in counseling people to warn people about the outbreak of the disease and also encourage the people from 20 years to 100years to do the screening. This virus is similar to that of HIV and people should get screened for its presence. When one is found with HIV, it can be treated immediately and it cures therefore you can to live for over 40 years. They should tell people that when you are diagnosed with cervical cancer at an early stage it can be treated to heal. We just need to be counseled because someone can be scared for no reason but when they get examples from people who were once diagnosed with the disease but now they are living health for 30years or 50 years. So, this is the same case with this cervical cancer virus, when it is discovered earlier enough it can be treated and one lives for more years. **[FGD_002]**

Bringing women together for awareness on cervical cancer screening is crucial. Assuring them of medical care if diagnosed, like with HIV, will encourage participation.

I had the same idea of bringing people together to inform them about the importance of testing for cervical cancer and also the dangers of the disease. These people should also be given hope that they will receive medical care when tested positive for cervical cancer as it is always done on HIV test. This will motivate women to participate in such programs. **[FGD_006]**

Raising awareness about cervical cancer's dangers can motivate people to get screened, especially when they realize its fatal consequences.

I think people need to learn more about the dangers of cervical cancer in order to be motivated because when they hear about the death of a colleague due to the same disease, they can be forced to participate in cervical cancer screening. **[FGD_003]**

Participants recommended that cervical cancer screening should be mandatory for pregnant women attending antenatal care at government health centers. They discussed that many avoid screening due to discomfort, but enforcing it as a requirement can ensure higher participation and early detection.

Something else mostly to the women who give birth from health centers, I think there should be put an iron hand on them as they go for antenatal care services in that everyone who has never been screened for cervical cancer. Some say that they cannot undress before the health worker when the pregnancy is not yet due and they deliberately refuse and decide to look for the private health centers. I suggest that the health workers in the government health facilities should make it a principle and a must because there are some people… to us, we are talking about women, when they are not used on an iron hand, they cannot act and when you decide to be kind on them, still you cannot manage them. Let it be made a rule for the women because cervical cancer is high in them. It should be made mandatory that whoever comes for the services should first be screened for cervical cancer and then they come back for antenatal. In that case if they were like twenty women, when the two of them run away at least the remaining eighteen should be screened. **[FGD_002]**

Participants discussed that making cervical cancer screening mandatory, like COVID-19 vaccination, would ensure higher participation. A screening card could serve as a requirement for accessing maternal healthcare services.

Making cervical cancer screening compulsory like COVID-19 will be a great opportunity to us. Covid-19 was also compulsory however some people would dodge to receive the vaccines but it was a must for every person because immunization card could act as a password medication. So in order for a woman to receive health care services such as childbirth, cervical cancer screening card should be a password. **[FGD_003]**

Participants advised that healthcare providers should handle clients with care, as past negative experiences can discourage future screening. they suggested that a friendly and supportive approach ensures women feel comfortable and willing to participate again.

They should handle clients with care for example; according to the different methods used to screen our colleagues, one of them fear to be screened again because of her past experience but if she had a friendly treatment, she couldn’t hesitate to participate in cervical cancer screening again. **[FGD_003].**

Healthcare providers should treat mothers with care and respect, as a positive experience encourages more women to seek cervical cancer screening

Just like my colleague has said, I would also suggest that the healthcare providers should handle these mothers with care not being rudely because when they are handled well, they will definitely influence others to go for the same service. **[FGD_004].**

Participants mentioned that sharing personal experiences can help change perceptions and encourage women to participate in cervical cancer screening, showing it’s harmless.

I think through sharing personal experiences by people who have ever undergone cervical cancer screening just like our friends have explained that the process is harmless, can help to change women’s perceptions towards screening. **[FGD_003]**

I could think that since the health workers attend to the clients on individual basis, it could be important to brief the person and emphasis confidentiality. This will inspire others to also participate in the screening. **[FGD_003]**

Women must take responsibility for their health by willingly getting screened for HIV and cervical cancer. It’s essential to know your status for early detection and treatment.

Me I would say that it is our responsibility as women to go to the health worker for instance for pregnant women now it is a must to check for HIV and cervical cancer so we should determine our selves not to say that I won’t be checked but rather decide to go and be checked so you can know your status. **[FGD_004]**

A free, designated screening center is essential, as many avoid checkups due to cost.

A place should be identified for screening and be completely free because people don’t want to put in their money especially for checkups. Is has happened to me before, I ignored a sickness and it got worse until I was taken to several hospitals but later I got better so I am speaking from experience. **[FGD_004]**

1.3. **Barriers to Participation**

Majority of participants highlighted barriers to cervical cancer screening include misconceptions, fear of discomfort, and privacy concerns. Many women hesitate due to lack of awareness or discomfort with the procedure, leading to low participation despite its importance.

“…you find that you can advise a fellow woman about screening for cervical cancer and they end up asking you that do they first anesthesia you? Because of being ignorant yet they would have loved the whole idea, also women do not feel comfortable opening them in their private parts and even looking at the person, she already has about 5 children yet screening has no issue per say. Actually, if you had said that we have a discussion today accompanied with screening you would not have any participants.” **[FGD_001]**

“When the women hear about screening for cervical cancer, they relate it to giving birth of a child associating this to too much pain although when I went for screening it did not hurt at all. Actually others.” **[FGD_001]**

“Actually, others say, if at all they go screening for cervical cancer and by mistake the cervix is damaged leading to other complications hence leading to the delay for us to seek for screening.” **[FGD_001]**

“...then another person will say the instruments used while screening is painful, that alone would discourage others because there is not any pain they feel yet and thus they would rather leave it at that.” **[FGD_001]**

“Other people ask how the screening is done, and it is explained in a way one is giving birth by lying flat then do the screening, they go ahead to say “they cannot” sometimes these statements are made by women who have given birth before.” [**FGD_001]**

“I think the method being used is scary. Whenever you tell someone to do like this or do like that [participant demonstrates the widening of women’s legs during the screening process] and women with such things… by the way that is one of the reasons why some women no longer deliver babies because of such procedures of telling them to do like this or like that [participant demonstrates the widening of women’s legs during the baby delivery process] and they insert in her the hand. Those are some of the things that limit them otherwise that method they use of inserting the machine to open wide the cervix scare us away”. **[FGD_005]**

Participants observed that many women avoid cervical cancer screening due to persistent myths and misconceptions, especially the false belief that the procedure involves removing or harming the cervix. Despite receiving explanations, some women continue to hold onto this misinformation and often refuse screening unless they are already experiencing pain, which delays early detection and treatment. The following quotations explains more about this.

“In fact, most of the women fear to participate in cervical cancer screening because of the myths and misconception that the process involves the extraction of the cervix from its normal position. They claim that the method used can be harmful to the cervix since it is very delicate but I told them that cervical cancer isn’t screened according to their perception. So that is their fear.” **[FGD_003]**

“For the others they say, the cervix is usually removed and put on plate, until one is able to explain that that is not how it is but because of the misconception or what they heard is what they usually pick up so you try as much as you can to explain the whole procedure on how it is done, they end saying you can go me for one am not going anywhere. And also, others say since am experiencing no pain at the moment I will not go anywhere for the doctors to examine me yet.” **[FGD_001]**

“I would think what really stops women from going for cervical screening is the wrong rumors spread about how it is done, someone could choose to say it is painful as one of us said that the cervix is usually removed hence one could choose not to go and after all if one focuses on the brighter goal of what is important such rumors in regards to pain resent women from going for screening.” **[FGD_001]**

Fear of cervical cancer screening, particularly when women hear distressing stories from those diagnosed or others who have had negative experiences with screening. These accounts create anxiety and discourage many from seeking screening, preventing early detection and treatment. This is as quoted bellow.

“One of the most hindrance of women to going for cervical cancer screening is fear, this comes about especially from someone who has been screened before and they are not sick but worry comes in from someone who is screened and found sick. For a case like this, they even narrate to you how they are treated that alone scares away the women. If we can do away with this fear then we step into place to get screened backing on whatever comes out of the screening maybe that would at least be important.” **[FGD_001]**

Some women get scared especially when they get such reports from other people especially those that have tested or screening, they also get scared to go for screening services. There is a case of a woman who went for the test but after the test she became even worse until she died so when someone hears such they decide not to go for the test so people have different perceptions but they are mainly based on what they hear from those that go for the test. Others wait when they get so ill. **[FGD_004]**

Cultural beliefs, such as the misconception that cervical cancer is caused by witchcraft, prevent some women from seeking medical care. This denial of treatment can delay early intervention, which could otherwise save lives.

“I have a comment on cultural briefs and practices. A number of people hold a misconception that cervical cancer is a result of witch craft. They even denial a patient a chance to be taken to the health facility for medical attention because of the misconception yet in case of early medical attention, this person’s life can be saved.” **[FGD_003]**

Participants mentioned that many women remain unaware of cervical cancer, as those screened are often HIV-positive and assume they are healthy. This in the end becomes a barrier to accessing the service.

“What leads to most women not to know about cervical cancer is that most people who are screened are usually people that were diagnosed with HIV and hence they end up quoting that they are fully fit and well and thus they cannot know that there is cervical cancer that is actually dangerous.” **[FGD_001]**

“I think what is limiting us women from attending the screening, is that we do not know the signs and symptoms of this type of cancer to know whether I have it or I don’t have it, so we neither know the signs on how it displays, whether it makes me to pass out a virginal discharge or it treats me in a certain way, just like those signs that show that I got the cancer. This makes us lazy since we are always thinking that we are well. What we have to do is, always go to the health centers for screening and not to wait for the signs and symptoms. But due to ignorance about these signs and symptoms I think it makes us reluctant to do the screening because we haven’t seen any of them. However, when you get a fever, you easily rush to the health center.” **[FGD_005]**

… The other one is ignorance about the signs and symptoms of that disease sometimes one may have a very serious problem that is taking away her comfort but when she can’t share it with anyone. She is aware that she has a vaginal discharge that looks like spoilt milk but still she decides to stay quiet and keep around with it. This could be caused by the ignorance about it. **[FGD_005]**

Many participants noted that a common barrier to cervical cancer screening is the discomfort women experience when examined by young health workers, particularly those they perceive to be the same age as their children. This sense of embarrassment leads many to avoid screening. Discomfort is also heightened when the health worker doing screening is a student doctor or male, with older women especially reluctant to undress or expose themselves in front of younger female providers. This was explained in the following quotations.

“For me for the women I have had an interaction with, they usually complain that when they interface with the doctors let us say when they about 50 years and they go ahead to provide a health assistant in the same age bracket of the children they have , they find it extremely shameful to allow these young doctors to go ahead and screen them that is why you find some refusing to go for the screening but rather they first get infected if at all the pain is too much then they can go and screen.” **[FGD_001]**

“For me what I think that stops people from going for screening, for example I have seen a scenario where women run away from the queue at the health facility this is because some of these facilities use student doctors. For instance a women who is about 50 years cannot allow a young doctor examine them because they consider them their children thus leads up a situation where some leave the queue. Another thing is women are afraid of men examining them especially with the older years.” **[FGD_001]**

“Mostly those ones who are aged like; they always say that “I cannot undress myself for the young women; I cannot widely open my legs like that before them”. Women commonly have that thinking but as for me I have so far been screened for cervical cancer twice and am not in that bracket. They used on me the other method I told you about of using… and the other one of a pen and I didn’t get any complications.” **[FGD_002]**

“… Some women are shy and yet the healthcare providers in the health facilities are largely youth, there are hardly any adults and so they get shy of undressing for the young ones. I was also screened but I went while scared however much I was willing, I could ask myself “where am I going to hang my clothes?” You may find there young healthcare providers, but still they have to screen you because you have determined from your heart to be screened. So this comes our own decisions making that you may decide to do it however, there are some who may decide to not do it and keep around with the fear and shyness towards the young healthcare providers. That is also a challenge but when you are determined to… because it is the current disease that is affecting people.” **[FGD_002]**

“Just like our colleague has said that they tell you to put off your entire clothes, you might go when your inner clothes are not in a good condition so you fear to be ashamed before the health workers so it is easy to shy away from getting the service”. **[FGD_004]**

“… women do not want to go for screening, it is what I told you earlier on that they find it unpleasant undressing them yet they do not know what could happen. For example one asked me if the screening is not painful. I let them know that it is not painful and it has no issue. And then also a person may not know when they have to visit a health facility to screen for cervical cancer and it is also hard for someone to wake up and move to facility without any sort of body pain unless if they get any sort of infection underneath and they go through all the checkups to ascertain the issue.” **[FGD_001]**

“I think what scares most people from attending the screening is because they have a torch that they light in you as they put your one leg on one side and the other one to the either side. So when a woman is made to appear like that sometimes it makes her shy away unless when is so determined. However, we must be strong and go for the screening.” **[FGD_002]**

That disease did us a disgrace to attack in that place. How I wish there was a different method of screening they could use such as the one of taking blood samples because that place scares a number of women and when one of your friends just includes it in the discussion it appears as something so scary. **[FGD_002]**

It is alleged that cervical cancer screening is a painful process. This was witnessed when a friend refused to participate in the same program for the second time due to its painful process. Such stories discourage those who have never tested before to also take part in cervical cancer screening to also take part. [**FGD_006]**.

Participants discussed that persuading women to undergo cervical cancer screening is challenging since many dismiss it as a personal choice. Because the disease is internal and not visibly apparent, few are open to discussions, making it difficult to encourage others in the community to participate.

“Doctor we do not have any way especially for us that go for screening to persuade these others that have not been screened before “they say you go with your screening” the fact that the disease in internal not external, there just a few that allow to be told about the screening and they agree to it most women do not want hence you cannot persuade another person in the village to go for the screening.” **[FGD_001]**

Some women believe they are not at risk of cervical cancer because their Muslim husbands are circumcised, assuming this prevents infection.

“The other thing a woman could say after all my husband is Moslem since they are circumcised and hence, they are never dirty after all where will he have gotten the cancer that eats up the cervix, so because of that belief they would choose to stick to that instead.” **[FGD_001]**

Participants reported that long wait hours at health facilities discourage women from cervical cancer screening, especially when they have no symptoms. Many are unwilling to spend hours in queues without experiencing pain

“If at all the facilities, where we would go to screen for cancer of the cervix would have available health workers that would attend to us and they we leave than rather have the women going to the facilities and really taking a long time while they are there seated for a long time. A case in point one would ask why they would go spend time there in long queues yet they hardly even have body parts that ache. Problem when the health facilities are quite busy.” **[FGD_001]**

Lack of essential supplies like gloves in government hospitals creates a barrier to cervical cancer screening. Women who cannot afford to buy them may be denied the service, discouraging those seeking screening.

“Yes, doctor when you go government hospitals, for example I visited one but I was requested gloves, for someone who would go there without them will have to pay around five thousand Uganda shillings and then the other one who would go there without it would surely leave without getting the service.” **[FGD_001]**

Limited equipment at health facilities leads to long wait times, discouraging women from screening. Seeing a single machine used for all patients raises concerns and spreads hesitation, further reducing participation.

“Doctor for me what am thinking, when one visits a health facility and they get to notice that it is only one machine in use and hence it has to be used on all the patients that come in at the facility one could be scared and they can hence tell another person about such an incident because it is really time consuming that alone could lead to failure of them to go for screening.” **[FGD_001]**

Husbands or partners' disapproval of women attending screening services is a key barrier, preventing many from seeking cervical cancer screening.

“… I for one think some women are being stopped to go for screening for cervical cancer by their husbands, some men tend to be tough on the women and they condemn them for leaving home to go take part in screening services I would think it is an obstacle for some women.” **[FGD_001]**

“A woman may decide and go for screening but then she may think that when she tells the husband, he may not agree to go for the screening and therefore she decides to also stay with the disease so that they both be sick.” **[FGD_002]**

Participants discussed that in some cases, women's submissiveness to their husbands prevents them from seeking cervical cancer screening. They fear that asking for permission will lead to accusations of infidelity, as their husbands may not understand the benefits of screening.

“According to my observation, women are too submissive their husbands which sometimes affect our lives because if a lady seeks for a permission from the husband to carryout cervical cancer screening, he will automatically blame her for having acquired the disease from adultery. So they don’t know the benefits associated with carrying out a cervical cancer screening”. **[FGD_003].**

“Being too submissive to the husbands also denies some of the women a chance to participate in cervical cancer screening programs however I don’t mean that women shouldn’t be submissive to their husbands but matters concerning life is very important to everyone.” **[FGD_003].**

Misunderstandings between the husband and the wife affects greatly our decision to undergo the screening. **[FGD_004]**

“In addition to what my colleague has said, there are women who cannot be allowed to come for such meetings to listen to what is being taught, their husbands prohibit them from attending such things so if there is no proper understanding, such a women will not know what is happening or the services available in the community, but if there is corporation between the partners health standards will be promoted.” **[FGD_004]**

“You may be having your husband… some men are scary; you may tell him that I want to go for cervical cancer screening and he will be like [eeeh] this woman has cervical cancer! That one too is scary. In most cases the men of these days are too adulterous and when you think of telling him and you feel like, what if I tell him and he thinks that I have cervical cancer and he drops me. So you get scared of going in for the screening and telling him and therefore you stay without having gone for the screening because of fearing to tell your husband about such [a muslim is heard in the background making a call for prayers]”. **[FGD_005]**

Participants reported that single mothers often prioritize household responsibilities over cervical cancer screening, fearing that taking time off could affect their ability to provide for their families. Additionally, many women struggle to make time for screening due to demanding work schedules, leaving little opportunity to visit larger health centers. This is as quoted bellow.

“Someone might say that I work from Monday to Sunday so I cannot waste time in going for the screening. When I spend like three hours without doing my house work I can even fail to eat. This is common among the women who are single mothers. Someone may frankly tell you that with such things I cannot but when she has her own reasons. She may say that if it is about screening, I do not have its time because if I take like two or three hours without working, I may not eat and so she may keep around on that point.” **[FGD_002]**

“Someone might be at the workplace and she fails to get time for her to go for screening claiming that she only has time for working. She may totally fail to find time to go to the health center. She goes in the morning and comes back at night to sleep and when she wakes up in the morning, she leaves for work so thereby leaving no space for meeting her. Remember, you have to go to a big health center because you can’t go to the clinic for screening. When she gets fever, she simply go to the clinic and get some medicines that make her well. So, by the time she goes to Kawempe or to Kawaala or anywhere else… people are not easy.” **[FGD_002]**

“Some women are always occupied with too much work to participate in cervical cancer screening. They take work as the first priority.” **FGD_003_03_10_2024 CACX-URBAN.**

The domestic activities leaves them with no space to participate in cervical cancer screening. **[FGD_003].**

Lack of confidentiality, both from health workers and other women, often spreads negative rumors that discourage participation in cervical cancer screening. Similarly, participants reported that healthcare workers sometimes share confidential test results with others, leading to community-wide dissemination of personal health information. This breach of confidentiality erodes trust and discourages individuals from seeking necessary medical care.

“Basing on what others have said, you realize that both health workers and other women do not keep the confidentiality of people’s information, for instance in that treatment camp at the Buganda County grounds a lot of information circulated of undressing women during the screening exercise so many did not turn up because of such information, those that went were not prepared because they were telling them go right now. When someone brings a rumor, for them they might be well yet the rumor might limit someone who actually could been the disease. It should be like in a confession room, what you tell the priest remains there. You come out and say your prayers then the other person also goes. This will help others to also go for the screening service”. [**FGD_004]**

“When we went for that checkup, it was like an emergency, the health workers were putting four women in a room so there was an instance where they told an old lady to put off her clothes and the health workers realized she did not have an underwear so one of them shouted “you don’t even have an underwear?” so she felt ashamed”. [**FGD_004]**

“… I also agree with other colleagues; the health workers don’t maintain confidentiality concerning the clients’ details. Cancer screening room is a secure but sometimes, the health workers share test results with our friends who recommend us to them for screening, who in return spread the same information to almost everyone in the community. So we request the health workers to maintain confidentiality because when you do so, we shall also have confidence to explain our health conditions to you.” **[FGD_006]**

Now days people are also disinterested in seeking health services, you can tell people that there is a health service that is going to be given in the community, people will tell you that “let those that have time go for it” yet those that say they don’t have time are very victims of such diseases. **[FGD_004]**

“In addition to my colleague, I would say that nothing should stop us from going for the screening for instance women who give birth through caesarian section go through a lot of exposure and pain but they do not stop giving birth so if you want to know the status of your life nothing should stop you and I don’t think I would come out of the room and I start narrating stories of what has happened in the room.” **[FGD_004]**

A cervical cancer diagnosis often elicits intense fear and anxiety, sometimes surpassing the distress associated with an HIV diagnosis. This heightened emotional response stemming from the perception of cancer as a particularly severe and life-threatening illness was a perceived barrier to cervical cancer screening.

I have been screened this year but what I know is that when someone is told that they have cervical cancer, they first get scared because cancer is a scary disease. When we got screened, there was someone who was identified and she screamed a lot thinking that life is done, [participant speaks as she laughs] but the health worker took her in a certain room and they counseled her and made her start the treatment. But the truth is that when you are told that you have it, you really get scared, actually you get more scared than a person who has been told that they are HIV positive because I think this disease is more dangerous than HIV. **[FGD_005]**

Many women lack awareness about cervical cancer, including its signs and symptoms. Health workers often advise women to undergo screening but may not provide detailed information about the disease itself.

“That is what I told you about. We the women are not so much aware about cervical cancer and therefore, we are not aware of its signs and symptoms [all participants repeat the same word] “we do not know them”. Such as, when I have this, it’s a sign that I could be having cervical cancer. So it requires these health officers to continuously sensitize us because when we visit the health centers they just keep on advising us to do cervical cancer screening but they don’t tell us” [Inaudible background voices from the participants about the submission]. **[FGD_005]**

Women in Uganda often face unexpected charges for cervical cancer screenings, despite services being advertised as free. This financial unpredictability discourages many from seeking essential screenings, especially those with limited resources.

To add on that lady’s submission, you may think… just as can you see Wakiso, Kakiri. The health workers always advise us to go there that the services are free of charge but when you reach there, the healthcare providers tell you to pay some money. So a woman may come from here knowing that the service is free of charge [inaudible voices in the background] and she carries only her transport but upon reaching there, they are told by health workers that the free things ended. So you find that someone fears to go there because of their financial status. So there are some who have the capacity to go there but still there are some who do not have it. Most of the health centers have changed overtime, they may say that cervical cancer screening is free of charge and we all gather up there and then after that the healthcare provider will tell you to pay ten thousand shillings. Now, imagine when one had gone with only her transport. **[FGD_005]**

Preference of private healthcare facilities over public ones due to challenges such as long wait times and poor service delivery in government health centers. Patients often spend entire days waiting for free services at public facilities, leading those who can afford it to seek care elsewhere.

“To add on that, something that limits us from visiting the government health facilities is that everything in them is hard to access and that is why we commonly go the private health facilities because when one visits them she gets treated. But you may go to the barracks and spend a lot of time there as you are waiting for free services someone who went there at 7:00am may end up leaving the place at 6:00pm. So also the poor service delivery limits the people from accessing the health facilities.” **[FGD_005]**

Some participants emphasized that nothing would prevent them from undergoing cervical cancer screening, highlighting their commitment to prioritizing their health.

Personally, as a woman I would say that nothing can stop me from going for the service because this is my life not for someone else. I cannot base on people’s rumors because they can be false so if I want to know my status I have to determine and go for the test. [**FGD_004]**

Nothing should stop women from getting screened, just as they endure pain during a C-section to give birth. [**FGD_004]**

**Suggested improvement of cervical cancer screening.**

Participants suggested that women should dispel misconceptions about cervical cancer screening by seeking accurate information from medical professionals. Further, consulting doctors ensures proper guidance and encourages screening over relying on hearsay.

For us the women we can begin by doing away with the misconceptions surrounding cervical cancer screening from others but rather seek actual information from the medical doctors, when you get there the doctors can give you right information about what is supposed to be done and hence screening can take place than rather listening to hear say. **[FGD_001]**

Participants discussed that Health care should organize community health sessions to encourage cervical cancer screening. Also, offering incentives, such as monetary rewards, could boost participation and increase screening rates.

You the medical doctors should organize health sessions in the community as a way to persuade people to screen for cervical cancer and add incentives such as money for every one person that is screened people will turn up (mummers in the background. **[FGD_001]**

Maybe doctor there should be health talks that elaborate more on the signs that indicate that someone has contracted cancer of the cervix or time when we have to be vaccinated cancer for the cervix, we need to get through such health talks. **[FGD_001].**

For me am thinking we would get those health workers who would create awareness through the use of the mega phones as they put out information in regards to cervical cancer and also put on when that has to be done on particular days so that we can be able to go screen. **[FGD_001]**

“…They should get somewhere else just as you know that these topics requires thorough sensitizations and proper explanations so that one understand since to us we first experience something so as to appreciate its reality. No wonder, even when we go to the health centers when unwell, cervical cancer screening comes last after carrying out the other tests and realizing that they are negative. So, it comes at the end as a last resort, we are lazy about some of the issues.” [**FGD_005]**

We need to put up a workshop just as you have come, we gather up or they announce on the speakers that… just as you see here and in Nabweru in case there is a function, they always advertise such that those who are willing may come and learn. When we are back we teach the rest what we have been taught and you never know that someone you have encouraged may also pick interest to come. Some people may fear but when even they have reason for being scared but when they see that you are back, the get encouraged and they promise to also go. **[FGD_002]**

“I would request whether it’s under the government or under any organization that has started this should continue mobilizing people. They can do it from the health centers where we get antenatal care from and they sensitize women. They can still move to the different schools like secondary and high schools as they sensitize the students because the fact is, very few people understand this cervical cancer.” [**FGD_005]**

“I think they should put more effort; as we have gathered here, they should use a mega phone to notify women about the cervical cancer screening as it is done on family planning programs. When people attend such programs, they are fed with important information about their health which may inspire them to participate in cervical cancer screening. But the people who absent themselves from health gatherings, they may miss out such information since they mind less to listen to health programs on either television or radio” [**FGD_006]**

Participants reported that healthcare providers should first raise awareness and sensitize women in the community about available services, especially cervical cancer screening. Educating women, particularly those of childbearing age, about the risks of ignoring the illness will encourage participation and readiness for screening.

They should first call/ create awareness among women in the community and sensitize them on available services that they are planning to bring to the community rather than just coming and creating an emergency telling people to go and have a certain service, for example women in child bearing age group are at high risk of getting cervical cancer so if awareness and sensitization is done by healthcare providers then women will be ready to participate because they would have known the danger that is in concealing an illness. **[FGD_004]**

Participants mentioned that women can advocate for cervical cancer screening by employing a strategy of sharing real-life experiences and encouraging others to seek the service. Additionally, personal testimonies from those diagnosed and treated can motivate more women to get screened.

We can also advocate try to our other fellow women on how best to seek for the service and encourage them to seek foe the service as we give lives examples to them such as incase ne has been screened and found with it they can say they were screened and also started their treatment; this would force others to also get to know where they stand in this case. **[FGD_001]**

Participants suggested that women can benefit from regular forums to discuss health issues, including cervical cancer screening. health workers can facilitate these gatherings and arrange group transportation to screening facilities, increasing access and participation.

I think as the doctors created a forum where we are able to discuss about various issues is it possible for the women to converge and discuss more just like we have done today or possibly if you screen for cervical cancer at your facility, we can converge here after this exercise and get transportation to the facility so that we can be able to be screened, this can be achieved as long as you take lead in this the women could agree to this. **[FGD_001]**

Participants suggested that cervical cancer screening should become a requirement for job seekers, many would likely prioritize it in order to secure employment, thus increasing screening rates. This is elaborated more in the following quotation;

Doctor maybe something more that I could think about that could persuade people to come for screening for cervical cancer. In Uganda unemployment is on the rise, if at all it is made up as a law that whoever is seeking for a job should therefore first be screened for cervical cancer most would opt for screening since they need the job. [**FGD_001]**

In my opinion, it should have been done as it was done on HIV/AIDS that it was mandatory to remove a blood sample for its screening. The same should be done with cervical cancer in that whenever everyone in the age range that is eligible for cancer screening goes to the health center to get the medicine, they cannot access the pharmacy to pick it, its say they were told to go to ward five. When they go there, they should tell them to go to the first room so that the health worker crosschecks their papers. Remember, a health provider’s handwriting cannot be easily understood by everyone who is not a health provider and so if am not, I will not be able to understand it and therefore I will take the card to the first room as I was told and when the person in that room reads it she will easily realize that this person was sent here for the screening. This should also be done in the same manner they do it on HIV testing when we visit health centers. You may go without the intention of screening for HIV but the health worker tells you that let us draw some blood, and you may think they are taking it for testing fever yet she is going to test for all of them and they get to know your status and they give you the results later when you are done with the rest. So this also should be done in the same way in that when we go to health centers whether you agree or not they just tell you that lets us draw some blood and they test for it to know who has and who doesn’t have so that we don’t have to wait to first make people agree to it because I may decide never to go to the health facility for the screening and you won’t persuade me to if am not willing but when you screen me indirectly, it may give me hope and also take away the fears I had. **[FGD_002]**

I recommend the health workers to be the ones to organize them whether at the universities or any other institution such that whenever any patient goes to any health facility even if it is just a clinic, they are to first get cervical cancer screening before the health worker attends to what took her there. This is so because people are now running away from the big health facilities to these small ones but if they all work together and we all agree on doing one similar procedure to everyone that visits the health facilities whether they go to the small health facilities or anywhere else, they have to first be screened for cervical cancer. I think that would help to reduce it. **[FGD_002]**

Participants discussed that Government facilities could improve efficiency by dedicating specific days or sections for cervical cancer screening, separate from other health. This would streamline the process and ensure better focus on each condition.

For me I would think as the government facilities, there should be sections with unique numbers these areas could basically be put aside to screen for cervical cancer and the for those with malaria, sickle cells or any other diseases could have other days. **[FGD_001]**

Participants recommended a strategy of Using a brush-like device to collect cervical fluid could be effective for screening if properly explained. Testimonies from those who've undergone the procedure can reassure others, increasing participation in screenings.

The first time they came at C/U [*referring to the Church of Uganda*],they used to give us things that appeared like a brush and they could tell us to go and insert it to the cervix and do it like this [*participant demonstrates rotation using her hand*]and after we return them. I wonder whether that procedure can be applicable in this case of cervical cancer or it requires themselves to insert it. But I think that they can still use it on this cancer because it looks like cotton though it’s not cotton it is just like a sponge. It has no harm and if they are offered better explanations about such a method with some people who can testify its effectiveness whereby one explains to them that when she went, they just told her to insert something that looked like a brush and it had a sponge into her cervix and it collected some fluids. This can help these people get testimonies from those who once attended the screening that it has no harm. With this, I think you can’t fail to get like two hundred people out of the three hundred people who commit and decide to go for the screening. **[FGD_002]**

Participants suggested extending services for cervical cancer screening near to the community for easy access.

“When such programs are extended into the community, it becomes easy for a person to utilize that chance and participate in cervical cancer screening from the community having failed to move to the health facility.” [**FGD_003]**

Participants recommended for a designated day for cervical cancer screening in health centers to improve the uptake of the service.

“I wish they get for us a day and we get to know that on a certain day in the health centers is for women just as you can see for the pregnant women, they have a day on which they go for antenatal care. And we also get a certain day that at the barracks when the women can go for the cervical cancer screening.” [**FGD_005]**

**Perceived importance of early detection**

“I really urge fellow women to go screen for cervical cancer, this is because I know a person who I was with last week they are now on treatment but sincerely speaking me I was really interested because I think one needs to be screened, the person went to hospital with signs such as pain in the lower abdomen when they screened that they have that cancer they boosted them that they will be well and can actually be able to bare children, she thought that as soon as one gets this cancer, they cannot not be able to bare children anymore but as long as one follows the rules as guided , they can live.” **[FGD_001]**

“Cervical cancer is a deadly disease but health workers always encourage us to carryout cervical cancer screening. So, I argue my fellow women to carry out the screening as early as possible and when tested positive, we should start medication before cervical cancer rises to another level.” **[FGD_003]**

**Myths and misconceptions**

Majority of the participants believed that cervical cancer screening involves removing the cervix. This was reported in the following quotation.

For the others they say, the cervix is usually removed and put on plate, until one is able to explain that that is not how it is but because of the misconception or what they heard is what they usually pick up so you try as much as you can to explain the whole procedure on how it is done, they end saying you can go me for one am not going anywhere. And also others say since am experiencing no pain at the moment I will not go anywhere for the doctors to examine me yet. **[FGD_001]**

Even the friends we interact with as I have told you that people used to claim that the cervix is removed from its position during screening process, if I hadn’t witnessed the screening process, I would still have fear to participate in cervical cancer screening. **[FGD_003]**

I once heard that when you go to the health center, they insert a hand and remove the cervix. That is what I have ever heard of though it scares away people. **[FGD_002]**

“One day there was a lady I was interacting with but she told me that she can’t accept to participate in cervical cancer screening. When I asked her for the reason, she explained that people say that during the screening process, the cervix is extracted from its position and put aside. I responded that cervical cancer screening is not done in the way she heard people saying”. So outside they are so many ladies with fear because of the same perception that cervix is extracted from its position during the screening process. **[FGD_003]**

In fact, most of the women fear to participate in cervical cancer screening because of the myths and misconception that the process involves the extraction of the cervix from its normal position. They claim that the method used can be harmful to the cervix since it is very delicate but I told them that cervical cancer isn’t screened according to their perception. So that is their fear. **[FGD_003]**

Participants perceived that family planning methods, like the three-month injection, can affect menstrual cycles and potentially contribute to health issues, including cervical cancer.

I put the blame on the different family planning methods we always use. For example, I use a three-month injection as my family planning method but whenever I use it, I take about three months without experiencing my menstruation periods however, I renew my injection experiencing my menstruation periods. But there is that person who renews the method immediately after the recommended period of the current family planning method even before experiencing her menstruation periods, yet it is health for every lady to continue using such methods after going into her menstruation periods. For my sake, I can take almost two weeks to a month experiencing my menstruation periods in order to release the waste I had kept inside my body. So I conclude by saying that use of family planning methods can also lead to cervical cancer. [**FGD_003]**

**Addressing myths and misconceptions**

The participant advised that women who have undergone cervical cancer screening should help dispel myths and misconceptions in the community by sharing the reality of the screening experience with other women.

I think it is the duty of us that have gone for screening before to be able to explain to these people that when you go for screening the open up your private and bring closer the cervix to be examined however the sterilizer used that is the spirit hurts a bit because it is meant to kill any sort of germs that is the information we are meant to provide to others and hence the screening does not hurt. [**FGD_001]**

Participants recommended providing more counseling to women as a way of persuading and encouraging them to undergo cervical cancer screening.

They have to provide counseling to us so that they capture our minds and heart so that we put trust in them. **[FGD_002]**

Women also mentioned the need the importance of not paying attention to information from the community which is negative to cervical cancer screening.

I think we should pay a deaf ear to the words that divert us from participating in cervical cancer screening and also give our lives the first priority by accepting to be screened. You may even find that the people with propaganda, “the cervix is extracted from its position during the screening process” have never participated in such services. So, it is everyone’s responsibility to cater for her life since it can’t be shared with anybody. **[FGD_003]**

# Social network and family influence

Women pointed out fear of rejection and the impact on family dynamics often prevent women from seeking cervical cancer screening. Some worry that revealing a positive diagnosis to their husbands may lead to abandonment or family breakdown, making it difficult to open up about their condition.

*“*You may decide and go for the screening but when they find you with the cancer it means that you will suffer with it silently without telling your husband. You may tell your husband and he leaves you thereby leading to the destruction of the family. He may say that, you will spread it over to me forgetting that he sleeps with you daily. It is not easy to package that information when taking it to him.” **[FGD_002]**

Women reported fear of misunderstanding within the family, especially with partners, discourages women from seeking cervical cancer screening. Men may question where the disease came from, unaware that they could also be a source of transmission, leading to tension and possible conflict in the family.

“My idea is, you may get some misunderstandings in your family. My understanding of men, they can pretend. You may come to him in a polite way and he will to start ask you different questions like, where did you get the disease from? Where did you go, how is the disease contracted since he doesn’t know how the disease is contracted not knowing that he could be among the spreading agents that brought it to you. He may forget that in the fast place as he is asking you the questions which may cause the misunderstanding in the family and things do not end up well.” [**FGD_002]**

# Fear of diagnosis:

Participants mentioned that fear of a positive diagnosis and the potential consequences, often prevent women from seeking cervical cancer screening. The worry of how to handle the news and the stigma attached to it creates a mental barrier, leading many to avoid screening altogether.

“Its fear that scares us from screening because I be fearing that what if I get aware that am sick when I tell my partner he will […] so it is our thinking that limits us from leaving our homes to go for the screening. We fear that what if I tell my partner that I have cancer and he leaves me or else he might ask me where I got it from or he may say […] so it is always our minds that create in us fear and we fail to go whereby I fear to go and I am told that I have it, so I decide not to go there. That is what limits us.” [**FGD_002]**

Some women reported fear of painful treatments and misconceptions about cancer treatment’s effectiveness led some women to avoid screening, opting to trust in faith instead.

Fear; people tell us that the machine used to treating cancer patients is very painful because it is an electric machine so that scares us. They also tell us that even if you get treatment cancer does not heal so you will finally die. So, some women say let me just stay home and trust God to keep me save rather than going into a machine. [**FGD_004]**

# **Attitudes towards cervical cancer screening**

# **Cultural influence on attitude**

Participants reported that cultural beliefs, such as attributing illness to witchcraft rather than medical causes, can hinder women's participation in cervical cancer screening.

Someone might be having a co-wife then they get sick may be of none stop bleeding then they will think or people can her that it is your co-wife who is bewitching you yet it could be cervical cancer affecting her so cultural beliefs impact women’s participation in screening. [**FGD_004].**

People are affected by different diseases but the mindset of witchcraft keeps people from seeking medical treatment simply because they believe that someone is bewitching them. [**FGD_004]**

Some women reported that cultural beliefs often attribute prolonged bleeding to witchcraft, which delays seeking medical care and cervical cancer treatment among women.

When a woman gets an illness of constant blood flow usually, they say that it is witchcraft yet in most cases it is a cancer killing them yet they could go and get treatment in a hospital, this is about culture, we tend to consult from our fellow women and do not go to health facilities. [**FGD_004]**

# **General awareness levels**

Participants attested that awareness of cervical cancer exists, but participation in screening remains low due to mistrust, misinformation, and skepticism about the benefits of free services.

Me I observe in women especially in our community do participate much in such awareness and health talks because if a woman wants really to know the status of their lives, then they will go for the test. An example is when AMBSO puts up camps there at the hill […] during the surveillance activities, we tell women to go for the services but they ask where they gain from, they say that those people just waste their time, they sell people’s blood. AMBSO is giving a free service but still they don’t go for the services given so even we tell them about the cervical cancer screening services they will not respond positively, they attach a lot of fallacies around the services given so women’s participation in such services is a heart issue if someone decides to participate then they will participate*.* [**FGD_004]**

Participants highlighted that majority of women lack awareness of cervical cancer, as it is rarely discussed, making it difficult to gauge their understanding of the disease.

It is also hard to estimate what the women think if they are not even in such group discussions, they don’t have any awareness of such a disease so even when women are discussing you cannot find them talking about issues of cervical cancer because they don’t know. [**FGD_004]**

**C-FACTORS INFLUENCING CERVICAL CANCER SCREENING**

**Fear of diagnosis**

Fear of a positive diagnosis discourages some women from participating in cervical cancer screening, leading them to avoid testing altogether.

The other thing, there are some people who have a saying that “[ahaa] let me stay with the diseases I have they are enough, I don’t wish to reach there and am told that I have cervical cancer”. So someone may keep like that when they are committed not to go for the screening. [**FGD_002]**

Sometimes it is fear, you may approach someone telling her to go to the health center for the screening of that disease and they fear thinking that what if she reaches there and after the screening, they tell her that she has it, so they get scared and say that she will not go. [Inaudible voices in the background]. [**FGD_005]**

Women mentioned fear of a cancer diagnosis and concerns about dying and leaving family behind prevent some women from seeking cervical cancer screening, leading to delayed treatment and worsened health.

Someone might fear that what if go to the health facility for screening and they tell me that I have cancer? What will I do, who will I leave my children with? We even think if they give me positive results. I might die so under such circumstances the illness worsens if someone contracted it. [**FGD_004]**

Participants highlighted lack of symptoms, fear of discovering unknown illnesses, and financial constraints deter women from seeking cervical cancer screening, especially when faced with skepticism from their partners.

“If someone does not feel any pain in his or her body, it is very hard for them to decide to go for a test. They even fear to be told of illnesses that they did not know they had. This coupled with financial constraints, when you tell your husband that I want to go and check for cancer of cervix, then he will ask you “what is wrong with your cervix, me I see you well.” **[FGD_004]**

Participant reported intense fear and discomfort with the screening process, especially the equipment used, can lead women to avoid cervical cancer screening, sometimes resulting in them giving false excuses to avoid the procedure.

Mine looks similar to that one’s. There is usually intense fear because those things… to me it was the first time and then the second one but still that machine they insert in you, it really makes you feel uncomfortable. To where I was, a certain woman told the health worker that she is in her periods and that she won’t be able to be screened because she isn’t ready but she wasn’t in the periods rather scared of the machine. [**FGD_005]**

Still to add on that point, Fear plays a big role. One day I was at Kawempe and I had committed to be screened because they had… but then women who went there came out and saying “[hoo] the machine is too painful” then everyone started saying that “am in my periods” and we ended up not being screened but it was caused by the fear that we didn’t to get screened [participants laugh]. **[FGD_005]**

**4.1. Social economic impact**

For example, a woman with some money, that is usually a plus for them they do not get much difficulty like the one without enough financial support most of the things done are easy go for them. **FGD_001_27_09_2024 CACX-URBAN**.

**Religious belief influence**

Some participants attested that religious and cultural beliefs, in form of misconceptions can discourage women from seeking screening.

“The other thing a woman could say after all my husband is Moslem since they are circumcised and hence, they are never dirty after all where will he have gotten the cancer that eats up the cervix, so because of that belief they would choose to stick to that instead.” **[FGD_001]**

“We have our dear friends the born again they have that kind of mannerism as they keep praying, they quote that “for all sickness to be healed” some few people would choose not to go to a health facility but rather trust those words.” **[FGD_001]**

God should forgive me but the born-again have done a lot to kill people. If a person is at all times in church seeking for only prayers… sincerely you may look at someone who is critically ill and everyone can clearly realize the sickness however much they are not doctors, and they keep on telling her that she will be well, she doesn’t have to go to the health center rather pray day and night. The born-again have played a big role in that area and by the time someone is taken to the health center, it’s too late and she is severely ill. We have experienced a number of such cases. **[FGD_002]**

“Also for a man that is Muslim the religion plays a big part in deciding because all men are circumcised and the man is left since he is circumcised they cannot have it yet this is a virus carried on , this could as well hinder chances of a woman to go for screening*.”* **[FGD_001]**

Something I have thought about unless the born gain there is a religion for the Muslims those believe that a woman must only be seen naked by their husband and other men cannot openly see her private hence leading to failure of some women to participate in cervical cancer screening. **[FGD_001]**

The born-again play a big role in that aspect. Someone may tell you that these medical services won’t heal you but Jesus can heal you. When things do not change, instead of telling someone that the prayers have failed to work and they advise them to go to the health center they instead insist on praying. They may even find someone who is in a critical state inside the house and they just offer a prayer but never advise that person to go to the health center. **[FGD_002]**

“There are some people in religions that do not understand what they believe and they refer to anything happening to being a devil’s action. Someone may say “how can a normal person like me undress before a health worker to screen me? That can never happen” so she reasons the matter with her own ideology in such a way.” [**FGD_002]**

“If there is someone who is a born-again here am really sorry, however am also a born again. The science of the earth moves hand in hand with the heavenly science and vice versa. For any saved born again, when you get any disease, please visit a health worker to get some medicine and thereafter come back and pray because those things walk together. When you get the medicine, come back and pray for it to work because when you drop one of those… actually that is why you hear that they are dying from there because they don’t go to the health worker since they expect to be prayed for. Those are the people you hear of that claim to be born again but when they believe something they do not really understand. The other thing… there is someone who has spoken of the clinics and she said that someone may say that “let me go to a private health center, I can’t allow to be screened…” In fact there some people who operate the clinics but when they are not health workers they only came to make money in this summer season and they can never tell you about the cervical cancer screening because they are after making money. There are the true health workers but also those ones who are after making money exist and they will never mention about screening you for cervical cancer since they are after making money.” [**FGD_002]**

Some religious denominations teach their followers to commit to prayers alone without seeking health care services yet it is God who also made health workers so it is important to seek medical attention as you pray. [**FGD_004]**

It is also important to note that some participants believed religious beliefs do not prevent women from undergoing cervical cancer screening; rather, some religions encourage women to address health-related issues. This was expressed in the following quotations;

“Doctor sickness is not dictated by any sort of belief, all regions for example Muslims, Catholics and born again they all fall sick so in this we just need to find a place where we can screen for cervical cancer.” [**FGD_001]**

There is no religion that can stop you from the screening because it is your life. When you table it with the majority, still it will be you to die. There is no religion that limits it. [**FGD_005]**

The Catholic Church supports medical checkups by encouraging women to get tested and organizing health camps for cervical cancer screening and other diseases.

For example, in catholic religion while at church, we are always encouraged to carry out medical tests especially women in order to know our health status. Sometimes the church even organizes health camps to test for cervical cancer, diabetes among other diseases. So they always encourage us. **[FGD_002]**

Muslims don’t organize health camps to carryout medical tests at mosques but they always encourage us to participate in screening. [**FGD_002]**

We are so much influenced by the religious leaders in that we quickly obey to what they tell us to do. So when they support these organizations in spreading this gospel, it may help us to see that the work is made easy if they preach it to us in the churches where we praise from. It will also contribute to see that we get involved. **[FGD_005]**

The misconception that a circumcised husband cannot carry HPV leads some women to forgo cervical cancer screening, relying on false beliefs instead of medical facts.

“The other thing a woman could say after all my husband is Moslem since they are circumcised and hence, they are never dirty after all where will he have gotten the cancer that eats up the cervix, so because of that belief they would choose to stick to that instead*.”* **[FGD_001]**

Addressing Religious concerns

Health workers through the church leaders should be given opportunities to speak in churches and other places of worship, where they can educate congregations about the importance of cervical cancer screening, address common misconceptions, and encourage early detection and prevention.

“If they can allow health workers to go to the churches especially on days of fellowships and they are given time to talk about cervical cancer screening, religious leaders usually know how to get people interested in things because people may not give attention to a health worker or a political leader but when a reverand father or pastor speaks people tend to listen and actually participate. An example is the recent immunization campaign where the religious leaders mobilized people and many went for the services.” [**FGD_004]**

Usually if the priest speaks of something in church or mosque, people that come out to oppose what they said a usually few. [**FGD_004]**

It is true; the religious leaders can mobilize women very well. This can be done after the service then the people are told to stay for 30 minutes and then the health workers can sensitize the people about these different diseases. [**FGD_004].**

Just adding my voice to my colleague, I also think health talks will help because women are difficult species, you can bring a health service in the community then they will ask you “what are going to give us if we test?”, how will they help us after testing? It is still my responsibility to treatment myself so let those who are sick go and be tested. [**FGD_004]**

The health educative talks really help a lot especially to those who are more concerned about their lives, yes, it is true that people are more attracted to something that gives them money but still if someone is more interested in a better health they will go for the test. Some of us who have aged we know what it means to go for these tests that we even pay for. People keep with illnesses simply because they are waiting for services where they are given money in return. [**FGD_004]**

**Perceived signs and symptoms of cervical cancer**

Some participants perceived over bleeding as one of the symptoms of cervical cancer among women. This is as explained in the following quotations:

“For one person I saw, they were bleeding too much beyond the normal bleeding while in their menstruation that is the time they decided to go and screen and see if they had cervical cancer.” [**FGD_001]**

“Repetition in the menstruation periods among women who are not currently using any family planning methods and they are okay, but when one went into the menstrual periods like this week, she skips like three days and after they go back in the periods. When one has such a sign, she might be having that cancer and is required to quickly go… because one has to go into menstrual periods once in a month.” [**FGD_005]**

“Still, I also think of bleeding, because whenever you see that you have got something that wasn’t previously on your body, like when you get repeating in the periods you get to know that there is a certain challenge and also the fibroids in the uterus are more likely to cause someone cervical cancer. I also don’t differ much from colleague because similarly I also had a friend who got a similar case. So, the fibroids too. When you get aware that you have fibroids, you should go for the screening and I think it is also among the signs that can indicate it.” [**FGD_005]**

“Constant bleeding for about a month or two months, I think that requires one to go for cervical cancer screening.” [**FGD_002].**

“I think that you may not bleed but feel pelvic pains in an abnormal way; this could also predict the presence of that cancer.” [**FGD_002]**

“Among the signs of cervical cancer I have ever heard of include bleeding and I have ever seen a woman who was heavily bleeding and when she went to a clinic in Mulago before it was phased out.” [**FGD_002]**

“There is a sign that I think can show that someone is developing cancer of the cervix though people don’t take it serious; when a woman get an issue of constant blood flow especially during her menstrual periods and usually when these periods are influenced by family planning methods, usually people who use family planning when they bleed it is a heavy bleeding. So if someone gets such signs it is usually a symptom of cervical cancer, for instance I had a relative who later died, she used to doubt that she did not have this type of cancer but after going for checkup she was told that she had the cancer so the issue of bleeding was constant until she died. So I got to know that when someone bleeds constantly even if they are not on family planning, that can be a sign of cervical cancer.” [ **FGD_004]**

“The signs am aware of include one having constant bleeding and upon the end of the bleeding, you may have a vaginal discharge which has a very bad odor. When the bleeding is about to stop, you may experience having a vaginal discharge that is very smelly. Those are the only two signs I know.” [**FGD_002]**

“I think also having pain in the fallopian tubes or in the pelvis area, is it pelvis area or the tubes? I guess all are the same. There are some ladies who can come and tell you that “nurse am having a lot of pain down here” [*participant touches the lower belly area*] and when you inquire whether they are about to go in for their menstrual periods they say that they have just finished them but still when they feel the pains every day. You offer them pain killers and when they are done still they come back with the same issue. So with such a problem one needs to go for the screening because it could also be also a sign that she has cervical cancer.” [**FGD_005]**

“The signs I know include, when a woman has a constant bleeding. That is the only sign am aware of.” [**FGD_002]**

Some participants perceived the itching sensation experienced by women as one of the symptoms of cervical cancer

“For me to be pushed to go screen for cervical cancer, I used to have itchy private parts that is when I went and screened for cervical cancer only to find there were other diseases.” **[FGD_001].**

“For me I think there some women who tend to have itchy private parts and they end up diagnosing themselves and hence buying some medicines to control t yet the disease keeps growing yet it is better to visit a health facility to be screened and know the truth.” [**FGD_001].**

“Sometimes you may be having a persistent infection that it causes you to have a vaginal discharge which makes you feel uncomfortable, itching, and a vaginal discharge that is smelly. So you appear before the health worker and explain it to him that am having such and such a complication. A health worker may treat you and he fail, so he advises you to go for cervical cancer screening to find out your status because sometimes it may first bring out the vaginal discharge when contracted and you may not realize that the cervix has some viruses since you may be treating the infection that doesn’t heal you could be treating it as it comes back.” **[FGD_002]**

“I think that also the urinals that are always dirty, when a woman enters and squats onto dirty place, you may find when someone has just urinated there, and she also urinates in the same place. This can cause women itching in the private parts and makes them to over scratch themselves and sometimes cause them to have a virginal discharge.” [**FGD_005]**

Some participants cited lower abdominal pains and pain during sexual intercourse as some of the symptoms of cervical cancer among women.

“I think in most case, you feel pain during sexual intercourse. You also experience severe abdominal pain.” [**FGD_003]**

“Some women choose to visit the health care facilities for cervical cancer screening after experiencing some of the signs and symptoms abdominal pain**.” [FGD_003]**

“Me I hear others say that if you have painful sexual intercourse with your husband or partner it can be a sign of cervical cancer.” [**FGD_004]**

Women mentioned the presentation of untreated sexually transmitted infections and their underlying effects as symptoms of cervical cancer.

“Women say that even when you are passing urine and it is smelly with other substances then it could also be a sign.” [**FGD_004]**

“Some women have watery discharge due to the effect of candidiasis or other infections. So, if a woman has candidiasis and it’s untreated it can also develop into cancer because the uterus gets affected in the long run.” [**FGD_004]**

“Me I would say these infections that we get, if not treated or unattended to then they can easily develop into cancer.” [**FGD_004].**

“I think among the signs includes the fibroids in the uterus mainly to the people who use family planning methods. Also, not having sexual intercourse just as the other lady said is a bad practice because one ought to have having sexual intercourse [*participants laugh*] because if you don’t have sexual intercourse, you are more likely to get fibroids. I have a sister of mine whose back pains […] the spinal cord was not functioning well because of not having sexual intercourse. So, when she went for screening, she was told that she had fibroids in the uterus and that this was due to not having sexual intercourse and also not giving birth to babies. I think that it is those fibroids that could sometimes cause us the cancer, because they could say that the fibroids are in the uterus. This could mean that even those fibroids may get wounds caused by the fibroids could also cause that cancer.” [**FGD_005]**

“It’s about the use of the different family planning methods because they are the ones that cause fibroids in the uterus and then I think it’s the fibroids that cause the cancer.” [**FGD_005]**

It is worth noting that a few women did not have any knowledge about the symptoms of cervical cancer.

“I have never heard or seen any signs and that is why I asked you about them.” [**FGD_006]**

“To me, I cannot point out a certain sign because when we go to the health centers, the health workers just tell us to go for the screening of cervical cancer. So, I can’t tell you that this causes it because whenever we go for the screening and it’s successfully done and they find me negative, they only tell me that am negative so still I cannot know what causes that disease.” [**FGD_005]**

**SECTION B**

**General results from the Key informant interviews**

**Study Title: Assessing the Perceptions, Attitudes, Knowledge (PAK), and Barriers associated with Cervical Cancer Screening among Women aged 25-65 years in Wakiso district, Uganda**

i) What are the perceptions and barriers of women aged 25 years and above to CxCa disease and screening in Wakiso district, Uganda?
ii) What are the factors associated with uptake CxCa screening among Women aged 25 years and above in Wakiso district, Uganda?

**A-KNOWLEDGE ABOUT CERVICAL CANCER SCREENING**

**1.0. Experience with Cervical Cancer Screening**

Most of the key informants interviewed possessed extensive experience in cervical cancer screening, with their years of involvement ranging from five to an impressive twenty years. Each informant brought a wealth of knowledge and insight, having witnessed firsthand the various challenges and advancements in the screening process over the years. From their extensive experience, they consistently attested to the fact that cervical cancer screening is often perceived as an uncomfortable procedure. Many of them emphasized that this discomfort can act as a significant barrier, deterring individuals from participating in regular screenings. This is indicated in the following quotation:

“The experience I have in cervical cancer screening I should say is of about seven years when am screening. One of the most things I have observed as I carry out this activity is that it is very uncomfortable for women. More so as you’re carrying out this activity. This is because as your carrying out this activity, you expose most of the most private parts of a woman. And this makes them uneasy. The good thing is ever since we got this experience in terms of training, we have helped women and this has led to an increase in number of women attending screening services. And this is increasing every now and then. It is also helping us know the health of women most especially with the cervix.” [**KII_001]**

“…. first, I trained in 2002 in the month of July then I went for the training of the cancer of the cervix in Mulago, meaning I have had experience of about 22 years.” [**KII_002]**

Findings revealed that cancer screening services are available at Health Center IV facilities and a few Health Center IIIs. Additionally, participants noted that referrals are provided for individuals who test positive.

“Cervical cancer screening is one of the services that we offer in the district and its mainly offered at health center IVs and a few health center IIIs and we receive lot of support from IDI so meaning we are well supported when it comes to cervical cancer screening however, when the screening is done, the cases that are identified are normally referred to Mulago cancer institute.” [**KII_003]**

Participants noted that most women are unaware of the importance of cervical cancer screening. Additionally, myths and misconceptions significantly influence their decision to seek screening services. This is as quoted below

“…… I have experience in cervical cancer screening. And in my experience, I find that women do not want to get screened with cervical cancer. Mostly driven by two factors. One is that people do not know the importance of screening for the cancer of the cervix. Then two there is a myth that when your screened with cancer of the cervix you will be found with the cancer of the cervix. So, in my experience very many people do not want to screen. Even the health workers and mothers and wives and girlfriends to health workers do not want to screen for cancer of the cervix. And usually those that are trapped to screen for example the HIV positive and those that go for family planning.” [**KII_004]**

All participants noted that they have one screening methods which involves using a speculum and …acid. This is as indicated in the following quotations.

“A woman lies on the bed after they have explained to her through counselling. Then, she is prepared. So we tell the woman that you are going to be put in a position similar to that of giving birth or pushing a baby. Then we lead them to a bed then we put a mackintosh. Then, the woman removes her clothes that cover her lower parts of the body. She then gets on the bed then flexes her legs and spreads them wide. After that, we clean her private parts or what we call perineum or vulva. Then after we get our different instruments that are designed to screen for cervical cancer. This includes the speculum and we insert it into her that helps us to open. It acts as a door in order to see the cervix. So, after we can be able to see the cervix, so we use a liquid known as acetic acid. It also depends on the concentration that you have at the facility. For me where I work the concentration is 98%. So with that concentration you get one mill liter and add 22 mill liters of distilled water. Then you get your clean cotton that you sterilized. Then you prepare then if you are able to see the cervix well maybe you also clean it if you find it having any dirt. Then after you dip a cotton into acetic acid that I have explained to you that you just made. Then you put on the woman’s cervix for about one minute. After one minute you remove it and read the results. How do you read the results; by looking at the cervix.” [**KII_001**]

“The methods are many we have visual techniques and these include VIA and VILI. VIA is Visual inspection with acetic acid. We use 5% Acetic acid and we put it on a woman’s cervix a fully exposed cervix for five minutes and observe for acetic whitening for one minute. That is when we get to see aceto whitening. Those lesions can tell us whether someone has cancer of the cervix. The other visual inspection technique is Visual inspection in Lugo’s Iodine. It is also called VILI. So VILI it’s the same technique the only difference is instead of using acetic acid we actually use Lugo’s iodine. And what you observe is not aceto whitening but you observe the mahogany brown changes of the cervix. Which represent lesions those with the cervical cancer will have visual inspection positive.” [**KII_004]**

**1.2. Factors that influence women to participate in cervical cancer screening:**

Awareness campaigns and health promotion efforts play a crucial role in encouraging women to undergo cervical cancer screening. Sensitization programs educate women on the importance of knowing their health status, which motivates them to participate. Additionally, the fear of illness and death further influences their decision to get screened.

“Hmm The things that influence them to go for cervical cancer screening, the first thing I think are the awareness campaigns mainly. The awareness camps that we give them. Where we tell them that it is very good for a woman to know about their health. The fact that women or even other people do not want to die. I think the sensitization we do the health promotion sensitization we do I think it is what has influenced them to continue coming. Maybe another thing that has influenced them from coming for screening is for the women who are on medication for them it’s a must that they have to be screened. Though it sometimes sounds as something forced but it has helped us.” [**KII_001]**

“Another factor the doctors have gone on to sensitize about cervical cancer screening for especially those women that are HIV positive. This has led them to come for the checkup, however before they have not been that active but now, they have started to come in plus also there has been a lot in the creation of awareness and now at least women have endeavored to come, also for the children that are on family planning they also come being that family planning causes some bleeding hence leads them to come and have the screening, so far so good at least this year I actually see that there has been some improvement.” [**KII_002]**

“First thing is knowledge. A woman is likely to screen if she gets to know that may be a neighbor died of cancer of the cervix but this is a preventable disease. Many of them will go and screen to avoid the eventualities of finding themselves in the late stages of cancer.” [**KII_004****]**

Cervical cancer survivors encourage other women to undergo screening. When identified early and referred for treatment, they motivate their fellow women to get tested upon their return.

“When they see survivors of cancer, because we have an implementing partner that takes on the survivors of different cancers but among that group the biggest percentage, I think 75% of them are with cancer of the cervix and these are people who were identified early and referred for treatment. So, when they get back through those clubs, they are able to recruit many other people to test.” [**KII_003]**

Findings revealed that education status plays a crucial role in influencing the decision to undergo cervical cancer screening. One participant noted that the more educated a woman is, the higher the chances she will go for cervical cancer screening, whereas the less educated a woman is, the lower her chances of getting screened. The participant said,

“The other is education status. The more educated the woman is the more chances to participate in cancer of the cervix screening compared to a P.3 drop out. Then employment status. Employment status should be different from social economic status. Ok let’s take employment status, women that are employed and are making some money for themselves are more likely to participate in coming health activities like cancer of the cervix screening compared to those housewives who depend entirely on their husbands for a lively hood. [Ahaa].” [**KII_004]**

One participant highlighted the number of children a woman has as a factor that might influence her decision to undergo cervical cancer screening.

“And the other factor is parity. I think parity, yes...I mean the number of children someone has had. The more the woman gives birth the more likely she is to screen for cancer of the cervix”.

**1.3. Barriers to cervical cancer screening.**

Male partner influence plays a significant role in a woman's decision to seek health services. Some men discourage their wives from undergoing cervical cancer screening, especially due to the belief that the virus is transmitted by men. This fear of being accused of infidelity may lead them to prevent their wives from accessing the services.

“Another thing I can think about is that even the men have an influence to women when it comes to medical services and caring for themselves. Those men who never went to school. Now if the wife tells the husband that at the hospital we were told to go and screen ourselves cancer of the cervix they may feel minimized. This is because as we are sensitizing, we tell them that this HPV virus is brought by men and this virus is the one that causes the cancer. Now, when the wife goes and tells them that are cheating and should let them go and get screened. There is a way it intimidates the man and they refuse them from getting screened. In the sense that if she is found having the cancer, she will think he is cheating or having other women. That is what I think.” [**KII_001]**

A participant mentioned that women experiencing gender-based violence may find it difficult to seek cervical cancer screening services due to fear, control, or restricted access to healthcare.

“Number two is sexual violence. Or what you would call domestic violence or gender-based violence. A woman who is struggling with domestic violence with her drugged husband will not have time to think about screening for cancer of the cervix.” [**KII_004**]

Participants discussed that a negative attitude from health workers could discourage women from undergoing cervical cancer screening. Additionally, they highlighted instances where an older woman might feel uncomfortable being screened by a young healthcare provider, which could further hinder participation in screening programs. This is explained in the following quotation:

“The attitude of the health care provider if they are rude to her, she cannot come back. The instruments we use to screen them are also painful if she felt a lot of pain she cannot come back. The position we put them in and ‘okwegala’ [ meaning opening their legs] for lack of a better word to be screened also makes them very uncomfortable. Even after seeing that the nurse is young, they end up deciding not to get screened. Others think after being screened for the first time they think it’s done.” [**KII_001]**

“The other is health care provider attitude. Women will be having the knowledge they hear from the radio of the cancer the cervix. The nurses alone and she is abusing everyone doing the simplest of the mistakes. So, very many women will get discouraged from getting the services from such people. So, improving the health care provider attitude in fact, if you have a poor attitude among your staff you may discourage many.” [**KII_004**]

Participants highlighted that the method of sample collection, which involves the vagina, may discourage some women from undergoing the procedure. Additionally, the lack of community awareness about cervical cancer screening serves as another significant barrier. This is voiced in the following quotations:

“One; is where the sample is picked from, because it is picked virginally, so many of these mothers if they are not in pain or they are not in labor they find it hard to really undergo virginal examinations so it is one limitation. Then two; is inadequate knowledge in some communities, communities are not well sensitized about the tests that are done and then three; some communities really don’t get to see cases of the cervical cancers so when they don’t see a case it becomes hard for them to take up a test because they tend to think it is very far away from them.” [**KII_003]**

“And in my experience, I find that women do not want to get screened with cervical cancer. Mostly driven by two factors. One is that people do not know the importance of screening for the cancer of the cervix. Then two there is a myth that when your screened with cancer of the cervix you will be found with the cancer of the cervix. So, in my experience very many people do not want to screen. Even the health workers and mothers and wives and girlfriends to health workers do not want to screen for cancer of the cervix. And usually those that are trapped to screen for example the HIV positive and those that go for family planning.” [**KII_004]**

“Most of them are ignorant, they just hear about this cancer of the cervix. We also have cultural norms; some find it culturally not fit to be checked thus they find it as a burden. Others it is about awareness and it is still very low, sometimes they first have to hear that a particular person was diagnosed with the cancer of the cervix. In that kind of situation this brings about 4 to 5 women apart from that many do not want to come. Naturally women do not want to be checked underneath hence they do not find it fit to go look out for a doctor.” **[KII_002]**

**Most of the women are ignorant, and they just hear about this cancer of the cervix.**

“Yes, the procedure is painful there is a chemical they insert there and that its painful and itchy. That is a myth. Acetic acid may be having a bit of sting but it is not as bad as talked about. I am not a woman but I have had instances where acetic acid touches my skin, I know how it feels so those are the three that, I think. The ‘kusitamila basawo’ that you may test positive when you screen, and that the procedures used are very painful.” [**KII_004]**

“When the woman does not want to be screened. She may think it is just things for the whites and they want to get money from us. The government wants to kill us like that. People say things that are weird. It is something that is aimed at helping her but she ends up thinking the government wants to kill her.” **[KII_001]**

Participants identified distance to the health facility as a major barrier to cervical cancer screening. Additionally, the choice of the preferred health facility may further influence a woman's decision to undergo screening. this is further explained in the following quotations:

“Apart from that, literally they are the same things like infrastructure. This I mean the roads, now some of the facilities like where I work, they are deep in the village. These roads make many stay home without coming for the medical services.” **[KII_001]**

“The other thing I can think about to the hindrance to cervical cancer screening is health care access. I may have knowledge that I should screen for cervical cancer but if XX hospital is the nearest hospital and I do not screen and you tell me to go to XX. Some will be demotivated to screen. So, lack of health care access is an important hindrance.” **[KII_004]**

Poverty was also singled out as strong barrier for cervical cancer screening emphasizing that when a woman has money her health seeking behaviors are good compared to when she does not have money.

“The first one is [aah] poverty. A broke woman who lacks money will not be able to get to hospital to screen the cancer of the cervix. So, the richer the woman is the more she will be having a healthy seeking behavior and the poorer the woman is the more limited she will be.” **[KII_004]**

**1.4. Perception of Age group to prioritize cervical cancer screening**

Results indicated that all participants agreed that cervical cancer screening should be prioritized among the adults. They discussed that at 22 years, it would be advised for a woman to start screening for cancer anticipating that she has started sexual intercourse. This should go up to 70 years when they have reached menopausal. This was elaborated in the following excerpts:

“When they have started to go into their menopause around 50,60 years. When they have started to get menopausal symptoms and she is advised by other people to also screen for cervical cancer. And that is when they will also remember that they should get screened.” **[KII_001]**

“So far as we try to sanitize the women we usually start with the girls that have started getting their periods. For all those that are young in terms of age and have not started sexual relations with men of course it is not necessary. However, from 22 years and above and actually those that are 70 years they do come around and get screened those elderly women, you get to a point where they say “my doctor please help and do the checkup”, my stomach started to ache help me check me because am a bit elderly you could find that I got cancer and it started eating me up, but with the ages of 22 and above we get them here as long as they have given birth before.” **[KII_002]**

“… the teenagers I think that age since they don’t really come so much in interface with health services they find it hard to really take on cervical cancer tests and then the elderly also of 50 years and above. It is those in the reproductive age that are very quick to take the tests.” **[KII_003]**

“The elderly. Yes, the elderly…Well, like I said the more you give birth to is the more likely a woman to interact with health care providers the more likely to get the knowledge and find it easy to participate in cervical cancer screening. Then number two is the other age groups is the young age 21 to 34 they grow up hearing about cancer of the cervix in schools and they present themselves for the screening of cancer of the cervix. Then they are told they’re not eligible for the cancer of the cervix. So she will wait until when she is 25 to 27 years to screen and then wait until. So the most unlikely age is the middle age.” **[KII_004]**

**1.5. Challenges in promoting cervical cancer screening:**

Participants noted that a major challenge encountered in cervical cancer screening is the frequent shortage of essential supplies at health facilities. Despite successful mobilization efforts through health education, the limited availability of materials such as acetic acid restricts the number of women who can be screened.

“The challenge I have encountered mostly is that things get finished from the health facility. And that is a very big challenge this is because you can get the person or even people after your health education and you have gathered people. You work on people and the requirements get finished. Like Acetic acid gets finished. You find that the people screened in that particular service are few. It is also a big challenge because you work and get tired and your attitude changes. As the nurse yourself your attitude changes you say am tired let me stop there. Those are the most challenges that I face. **[KII_001]**

The other challenge is sometimes the acetic acid gets done and it takes a long while before getting another and if at all another comes around, they cannot be able to get the screening service.” [**KII_002]**

Another significant challenge is the referral process for women with advanced cervical cancer. While early-stage cases receive immediate treatment, those requiring specialized care are sent to the cancer institute, where referrals are not always prioritized. Additionally, limited space at health centers further hinders service delivery.

“Another issue is when we send these women after trying to asses and work on them first time, there is usually a medicine we insert in them especially for the early detection. However, in case the cancer is advanced we have to send them to the cancer institute it is my challenge that they do not consider working on a referral so that they can be sent back. They at times make the woman move around back and forth with those follow ups it doesn’t make me feel good. At the health centers the space is still limited and the service would at least be brought to the other health centers such as health center 2, despite that we do these outreaches this service should be available so that when a woman comes in they are able to be worked on. You find that sometimes the women give in and end up coming to be worked on. If at all they do not get the service and this becomes a challenge to us health workers because it ends up looking like the service does not attract people.” **[KII_002]**

“Lack of supplies. I work at XX also but all the supplies we are using are either mobilized from donors or they have provided with funding partners. We don’t have almost everything for cancer of the cervix screening from the Government of Uganda. So when we get materials done from the funders and sponsors it’s a barrier to screen mothers**.” [KII_004]**

The major challenge remains the lack of sufficient awareness about cervical cancer screening. very six months. Majority of women may express interest in screening, but without consistent messaging, participation remains.

“The lack of creation of more awareness is still a big challenge. We need to boost it enough as they do more advertisements. Most of the times the women may get interested so at least every woman knows that with in a space of 6 months every woman is screened for cervical cancer.” **[KII_002]**

“Access; there are communities that are far from health centers so finding a service is hard for them. The other is knowledge gap, communities have lots of knowledge gaps about issues of health, and basically I think that is it.” **[KII_003]**

Misconceptions and misinformation within the community present significant challenges to promoting cervical cancer screening, leading to widespread doubts and reluctance. Such misinformation not only hinders vaccine uptake but also creates fear and skepticism around cervical cancer screening services, further limiting access and participation, especially among village residents. This is as quoted below;

“….. the recent misconceptions from community states that the vaccine injection administered to the children is the one responsible for spreading the cancer that is sounding so much and I heard it because I move and mobilize in the villages, those people in the village advise each other not take the children to get the injection because it spread cancer this also hinders people from coming to get the service especially those in the villages.” **[KII_002]**

“One; there is a lot of misinformation about cervical cancer and it creates disbelief where people don’t believe in those tests, they think maybe these people are bewitched so each time they see a case of cervical cancer they really think it is a case of witchcraft so that alone is a big challenge to convince numbers to come for those tests.” **[KII_003]**

A major challenge in cervical cancer screening is inadequate staffing. Additionally, there is a lack of government support and political will, including insufficient funding for awareness campaigns. The high cost of advertising cervical cancer screening on mainstream media further limits efforts to promote the service. This is further explained in the following quotation.

“Two is staffing. We have an oncology unit but actually it has two nurses. One is currently on leave and it has only been one and what if she gets sick and like I said attitude. Even if she has been having a good attitude or she has been well motivated if she gets over worked her attitude will change. So low staffing is a barrier. So number three is lack of support by the government of Uganda. Lack of political will. I would think advertising the cancer of the cervix screening by the government of Uganda. But if you to take an advert on NBS television today for that it will cost you as though you benefit from it. So those are some of the barriers we face.” **[KII_004]**

**1.6. Encouraging women to participate in cancer screening:**

Participant mentioned consistent health education on the importance of cancer screening and sharing personal experiences to build trust.

“I encourage them through health education, you teach them and stick to it. You tell them this thing is good and I also got screened got screened. And when you get screened and you get to know that you are ok it gives you peace of mind to know that your inner body is doing well.” **[KII_001]**

“One; we do a lot of health education especially within the health centers to those who come to our facilities, then once in a while we do health education in communities and then we also have those cancer groups like I told you we have an implementing partner that has taken on the survivors of cancer, they have very good mobilization strategies, they use drama, they use local radios, and once in a while we also have a radio talk show about cervical cancer as a department.” **[KII_003]**

“Well, we appeal to them to keep to the reason they had come for screening in the first place and the reason is because they are at risk of getting cancer of the cervix. And that if you ever get it early it can be treated without necessarily from stopping your work or normal life. And that the screening now does not stop you from getting cancer of the cervix five or three years later. And depending on which method you have used it won’t stop you from getting cancer of the cervix three years to come. Or, having to continue being motivated and to know the reason why you screened in the first place and screening only once does not help.” **[KII_004]**

A participant discussed a strategy of using recorded stories to build trust and attract more women to seek cancer screening services.

“[aahaa] we do have recorded successful stories. We have what we call; of course after patients consenting of course we take off identifiers from the success stories. We have testimonies who may be have been having stage one cancer of the cervix. Or stage 1a, 2 and we have done minimal surgeries on them. And they have actually healed though we are still following them up for about two years. They are still under follow up and we are sure they were having it and now they are ok. It’s a good thing and keeps women coming.” **[KII_004]**

The participant mentioned a strategy of encouraging parents to vaccinate their daughters at ages 9-10. Additionally, discussed engaging with girls, teachers, and matrons, while also working with village health teams to raise awareness and promote cervical cancer screening in communities.

“First and foremost, I encourage them to vaccinate their children for the girls starting with the ages of 9 to 10 before they get involved in sexual practices with boys. Secondly, we have tried to visit and sensitize and do health education talks about cervical cancer in schools as we talk to the girl children, their teachers and matrons as to hold to build a sense of understanding for the people about things to do with cervical cancer. We got to realize that if we do not get into school, we cannot get to what we want, so we go to primary and secondary schools. The other thing we have these village health team members that we empower with knowledge so that they can get into the villages and the help with sensitizing the women on how they can go for screening. Even when we do these outreaches, we still try to sensitize more about this cervical cancer screening.” **[KII_002]**

**1.7. Suggested areas for improvement**

Participant suggested conducting trainings for health workers to enhance their skills in cervical cancer screening.

“For I think it should be through making sure we teach all in all health workers facilities so that they are all skilled because they usually train a few health workers yet the facility has many.” [**KII_001]**

“I would like to add for the people in higher authority is to put in more effort in training other nurses they all learn this procedure because the experience they get from training schools is too little. And even they do not know the new information. Now they go training few nurses and it’s those they train that go ahead to screen and this makes them get tired which leads to the attitude I talked about. And when the woman gets to the nurse when they are already tired.” **[KII_001**]

“first of all we need more trainings for example me I stopped going for those trainings around 2002, then we had another small training a CME at the hospital. You find that these things to do with cancer keep changing thus we need refresher courses.” **[KII_002]**

Participant recommended a need to increase on human resource to have a smooth implementation of cervical cancer screening. they said,

“The first way is to increase on the number of the health workers, in that if today there is one the other day it could be another person so that they can be able to be alternated so that it is not just one person present because this becomes very tiring.” **[KII_002]**

“You begin by recruiting staff enough staff not be over whelmed by the other health provisions which are already being done. Such that there are those already existing for cervical cancer screening. If you have enough redundant staff which is a rear thing in Uganda. But if you have you can do an on-job training of the existing staff so that we can include them.” [**KII_004]**

Participants recommended an adequate supply of essential screening equipment. They suggested that these tools should be readily available in all health centers to ensure uninterrupted service delivery.

“The equipment to be used to be in place at least for example sets of the visual examination equipment such as the vaginal speculums to be in plenty and be given out in all the health centers and in all that has to be used in the cancer screening examinations to be in plenty like the acetic acid as well.” **[KII_002]**

“Two, is to provide the necessary equipment and material needed for cervical cancer screening in every out post of the health facility.” **[KII_004]**

Participants suggested for sufficient examination space to allow multiple screenings to take place simultaneously. One participant mentioned,

“Thirdly where they examine from, there should be creation of enough space when one is examining here another person can as well be examining elsewhere so that the patients hurriedly leave and hence the service does not delay the patients at the health Centre.”

The government should increase efforts not only in sensitization but also in actively convincing people to undergo cervical cancer screening.

“The other thing I think even the government should put in more effort…. It should put in more effort in getting different ways of sensitization. Not necessarily sensitizing but convincing people to screen. Just like the government put more effort in encouraging people to get immunized against different diseases they can also put in more effort to turn up for cervical cancer screening.” **[KII_001]**

“If at all we can create awareness slowly by also creating rapport with them, it will be easy to create awareness in the churches. The churches can actually help us create awareness, the religious leaders would not have any issues but because they also do not know, they stay there with thought of advising people to just go to the health centers thus we feel like they also need sensitization.” **[KII_002]**

To improve cervical cancer screening, Participants recommended a need for more community outreaches with major emphasis put on remote areas where access to health services is limited. He said,

“One; I think we need to reach the communities, meaning we need to do more outreaches because the people who come within the health facilities those are converted so we need to do outreaches that target communities that are far and not accessing services then two; I think we need to clarify certain things about cancer because people tend to attach a lot of witchcraft around cancer so you find that as health providers we need to do a lot of clarification of facts around cancer and then we need to do a lot of prevention because we have the HPV vaccine but the uptake is quite low, actually it is one of the most under-utilized vaccine and yet we have a lot of it, it even expires on us but parents don’t want their children to be vaccinated because of the myths around it so we need to do a lot of health education**.” [KII_003]**

“I will start with appealing with for political will. Whenever there is political will there is funding and the funding is so mega about cervical cancer screening. So when we get enough funding we will be able to reach out do out posts, do a number of medical camps of also cervical cancer. Number two is allocation of staff and funding. Number three is putting up effort to educate more about cervical cancer screening. You know it’s not an everyday thing it’s not like phlebotomy. It’s a particular skill. A lot of people get trained while still in school how to screen. We therefore need to get more effort with recruitment more staff and train them how to screen particularly. And I think we shall be good to go.” **[KII_004]**

**1.8. Integration with other health sciences**

Integrating cervical cancer screening into routine HIV care has been shown to significantly increase screening rates among women living with HIV. Additionally, participants suggested scaling the integration to family planning and immunization section.

“I think this way of integrating is already into play according to what is happening. People who come to the hospital for a refill of their ART drugs are always screened provided you’re a woman 25 and above years. Now that is a very good strategy because women are coming because they know they have to come for a refill for their medicine they take for survival. They come and do it at once. So, the strategy is already there and its being done.” **[KII_001]**

“I think those that are not on ARVs at the time when they are coming in for family planning services they can be screened. The fact that at that time they are not pregnant. So the way to integrate screening with family planning. Like we know in order to get any family planning g services we first get a urine sample to check if you are pregnant. In another way I think if you come to get family planning, we then we get a urine sample if you are not pregnant. Then we screen you for cervical cancer if you don’t have then they give you the family planning that you want. That way it can also work.” [**KII_001]**

“One; I think integration is happening but not to a far extent because when you look at the ART clinics it has already been integrated, and it is also in the immunization clinics. What is lacking is the outpatient so maybe we need to have a one stop shop center for all services such that in case someone has come for OPD services they are able to get any other services including cancer screening.” **[KII_003]**

**1.9. Perceived importance of early detection**

Early detection of cervical cancer is crucial, especially when the virus is still in its early stages. Screening allows for the identification of lesions, which, though not cancerous yet, indicate the virus is affecting the cervix. Early detection enables timely treatment, preventing the spread of the virus and offering a higher chance of healing.

“There are many importance of early detection. But what I think is more important is: early detection or screening for cervical cancer when they are still young. If you realize you have the disease of the virus has started to eat up your cervix. They can help you early because there is medicine. Am not going to call it medicine, but there is treatment that is unique in case there are lesions. The cancer of the cervix forms lesions on the cervix they look like patches. If they make those patches, it’s not yet cancer of the cervix but its showing that the virus is starting to eat up this part. Now how does early detection help us. If a woman screens early enough, the virus has not spread so it helps her to get treatment early enough. And it is the most important thing. If the people get this service early they can be helped and they get healed.” **[KII_001]**

Early screening for cervical cancer can prevent its spread and increase the chances of successful treatment. Delaying screening allows the cancer to progress silently, weakening the body over time.

“For me I have had the chance that all the patients we have screened and found out that the cancer is in its early stages, there is a small thing that we put we have realized that the cancer does not spread, meaning if they are screened while it is still early that cancer can be cured and it does not spread elsewhere but if you take long without being screened, that is the problem comes from and again n it goes on eating you up every other day. We should put in consideration that there is no much pain you feel but it just continues to eat you up based on the nutrients you have in your body.” **[KII_002]**

Cancer weakens the body, and without early screening, it can lead to the loss of vital body parts, like the cervix. Early detection is key to preventing such outcomes.

“Let’s put it in mind that when you have cancer your body weakens in that any disease can affect you directly. The other thing is that incase cancer is in your body, one can lose some body parts. For example, no one wants to die without their cervix yet they were born with it that would not be a good thing but since you may not have screened early enough one can end up without some body parts.” [**KII_002]**

Early detection is crucial for saving lives. Those detected early have better survival rates, while late detection limits the chances of successful treatment.

“If a client is really detected early, these are the people we are seeing as survivors because those that are detected late, we can’t save much because the survivors we are seeing were really detected early so meaning early detection is something very important and it can give us at least good outcomes.” [**KII_003]**

**1.10. Myths and misconceptions**

Myths and misconceptions about cervical cancer screening include beliefs that it is a scheme by the government or foreigners to harm or exploit women rather than a life-saving health service.

“When the woman does not want to be screened. She may think it is just things for the whites and they want to get money from us. The government wants to kill us like that. People say things that are weird. It is something that is aimed at helping her but she ends up thinking the government wants to kill her.” **[KII_001]**

Some women avoid cervical cancer screening due to myths, including fears that a positive result confirms a husband's infidelity or that healthcare providers are spreading false information for financial gain.

“I usually hear them say is that if they find out that you have this virus that causes cervical cancer they think their husband is cheating. Now, the fact that they think they will now if their husband is cheating now that will stop them. It is one of the myths that stop them. Another myth but it is always at a low percentage with these mothers around who I call playing with their life. They say that we want to spend money to show they are working and whatever they are saying is not true. So some people think what you are going to tell them is wrong information. And those are some of the myths women have.” **[KII_001]**

A common misconception in villages is that the HPV vaccine causes cancer, leading some parents to discourage vaccination, which hinders uptake of the service.

“The other thing could be us going back to our children, the recent misconceptions from community states that the vaccine injection administered to the children is the one responsible for spreading the cancer that is sounding so much and I heard it because I move and mobilize in the villages, those people in the village advise each other not take the children to get the injection because it spread cancer this also hinders people from coming to get the service especially those in the villages.” **[KII_002]**

A major misconception is that the HPV vaccine causes infertility, making some parents hesitant to vaccinate their children.

“The most common one I hear is about the vaccination injection they actually that when they vaccinate their children with the injection we shall prevent them from giving birth. About screening, they do not have much knowledge about it because they say they are being screened to see if they have the cancer or not so they do not have any misconception about that but rather the injection.” **[KII_002]**

Many believe cancer is caused by witchcraft or punishment from the gods. Others think it spreads through the air, leading to stigma and isolation of those diagnosed. These myths create fear and hinder early detection and treatment.

“People believe that all forms of cancer are witchcraft, people must have been either bewitched, that is how they could have got those long term illnesses and they end up dying then two; another myth is people think cancer can be gotten either in the air or they don’t understand the clear root of how someone gets the cancers so they believe if someone gets the cancer they should be isolated, they are stigmatized because others believe they might get the same disease through breathing the same air or something of the sort, the other myths around cancer is people believe it’s a punishment from either their small gods that maybe the gods are not happy with you that is how you end up getting a terminal illness like that one.” [**KII_003]**

**1.11. Addressing myths and misconceptions.**

Addressing myths involves reassuring patients about the safety and benefits of screening, clarifying misconceptions, and emphasizing the importance of early detection. This builds trust, encourages return visits, and promotes referrals.

“Now of course the women or all these patients at the time they are mentally sick, I start with counselling them that all that about to do has no harm but it will help you and me so that we can get rid of something for example one coming and saying doctor now you see me I have been bleeding for about 2 months yet I have been on family planning, so I want to do away with the issue that family planning has brought cancer thus by checking and confirming to them that let us take care of this disease but it is not cancer so that is one. Secondly, me I can persuade the woman to be interested by letting them know that for example “don’t you now see that you have been sick for a long while and we are always treating this, why don’t we screen and see if you have cancer?” so that I may know why do you often get issues inside there.so you first counsel and let them know the importance in it, thirdly you let them know that if we find this, this is what we shall do so this helps to boost their morale so that they are able to come back and even recommend another to come. You find that they will tell, you that I was also able to recommend this one to come as well basing on how you talk to them.” **[KII_002]**

Health education is actively carried out through a dedicated department, along with support from implementing partners who focus on cancer survivors. These efforts include publications and community sensitization to raise awareness about cervical cancer.

“We are doing a lot of health education, there is a whole department of health information and education so it does a lot of that both within the facilities and outside, and then we have implementing partners like the one I told you about that supports survivors of cancers, so those people do a lot of publications and health education and sensitization of communities about cervical cancer.” **[KII_003]**

**1.12. Follow-up and management strategies**

Cervical cancer screening involves treating lesions with a metal device that burns them. Women are scheduled for a follow-up visit after six weeks, with details recorded in their treatment book. Potential side effects, like bleeding or increased vaginal discharge, are monitored during these follow-ups.

“Now, if we screen and find them having those lesions, we give them a therapy. It’s the treatment we give them ok its like a special metal that we put on the cervix and it kind of burns that place. It provides a kind of heat that burns those lesions. So in terms of following up the women this is how we do it. In case we use that kind of metal we have used to treat the lesions. She has to come back after six weeks. So it is a must and we also tell them that we found lesions on your cervix now we have given you treatment but after six days come back. We document this in our register and also in her treatment book we also give them a return date and we record it also. We also tell them even the things she may experience from the treatment she is getting. Those things may be like bleeding a little or the vaginal fluids may increase or they may experience lower abdominal pain. She tells us those things as we follow up. Even on their card for cancer of the cervix has follow up dates.” **[KII_001]**

After screening, patients are referred to Mulago hospital for further treatment. The team has a contact to confirm with the doctor if the patient was able to access treatment.

“Like I told you before, we have our referral system here after screening here we forward them to Mulago hospital but we take caution to call the patient to confirm if they have been worked on so that is where we still have a challenge that after they have been worked on we cannot know much but apparently we now have one of the doctor’s number to confirm that they were able to receive that particular patient but remember that doctor does not work every other day.” **[KII_002]**

“Normally like I told you, we refer them to Mulago for further management because we don’t have a treatment center here yet so all our cases where we are even doubting are referred to Mulago for re-screening and those who are identified to be sick are also referred for management.” **[KII_003]**

The team maintains patient contact information and uses a diary system to track follow-up screenings therefore, patients are contacted when it's time for repeat tests

“We keep patients contacts and we have someone who looks through and we have a diary where we keep them and someone calls. For example, if I screened you may be three years ago with a PAP smear. Someone has a dairy and they check and they call; hello XX I think your time is up for a second or repeat PAP smear test. Can you please come or if you cannot come can you go to a place where you can do this again? That is how we keep up some times the hindrance is when someone is unreachable. The good thing is we encourage a good health care provider attitude. When someone has a good attitude the first-time experience when screening they will come, its automatic when they are expected to be treated well, they will come back.” **[KII_004]**

**B-ATTITUDE TOWARDS CERVICAL CANCER SCREENING**

**2.0. Cultural beliefs or practices influence on attitude**

Some participants believed that cultural beliefs do not influence attitudes toward cervical cancer screening. Instead, they attributed these attitudes to a lack of knowledge and literacy.

“…Haa I have not seen that in the perspective of culture for me I see it in the perspective of illiteracy. For me I think if someone refuses to screen for cervical cancer is because that person has not gotten to understand but not because of the cultural beliefs and practices.” [**KII_001]**

A participant noted that while cultural norms in some communities may make women hesitant to expose their private parts to healthcare providers, these cultural factors should not deter women from seeking cervical cancer screening. a participant noted,

“I have done most of my research and most of my practice in Buganda especially about the cancer of the cervix screening but I interact with many women from different cultures. The Sudanese hate opening their legs for a health care provider if they are not pregnant and if they are not in labour for that matter. As for the Baganda I think they are ok. The other factors will always determine how she will turn up her but when she turns up her culture will not affect whether she screens or not.” **[KII_004]**

Cultural beliefs, particularly those attributing illnesses to witchcraft, significantly hinder the uptake of cervical cancer screening in Uganda. Many individuals, especially in rural areas, consult traditional healers before seeking medical advice, delaying early detection and treatment. This reliance on traditional practices underscores the need for culturally sensitive health education and interventions to improve screening rates.

“uhuh, do you know that is one bad thing here in Africa? Culture has spoilt the medical field this is because so many of them you first have to get to them and tell them you know what you do not need to first go to a witch doctor but rather go to an actual medical doctor. So that they can check and think about something else, or else we can go to the hospital together with your “mumbwa” local herb in that according to their thinking they still consider that the “mumbwa” helped, were am I deriving you to culturally the women or people still have thought African belief that most diseases are brought about by witch craft .Even if someone one was disgonised HIV positive they are liable to getting cancer of the cervix but they could also wake up one day concluding that they have been bewitched . To start thinking this way they end up making up stories that my co wife bewitched me “ekikulukuuto” bleeding disorder that is why my husband cannot even come close to me because “nkulukutta” bleeding and I have spent 6 months without being with my husband, cultural beliefs have stake in disorganizing the health issues. Sometimes you need to end up working closely with the people where they usually go to seek help for example there areas that still have Traditional birth attendants (TBA’s).” [**KII_002]**

Participants discussed those cultural beliefs significantly hinder women's access to cervical cancer screening in Ugandan communities. Many women attribute health issues to witchcraft, leading them to consult traditional healers instead of seeking medical care. This reliance on traditional practices delays early detection and treatment of cervical cancer, underscoring the need for culturally sensitive health education to dispel myths and promote screening services.

“And in the villages there many. That “mulelwa” is responsible for many things, you can go there and they tell you that a certain woman went in for family planning services but even when they stopped using it there is a co wife of hers bewitching her. For such a person is the one you get in touch with so that you can have a conversation with, after having a chat with them and change how they think, they can now ably come to the hospital for screening and maybe diagnose them that they have the cancer, although when they are told that they have cancer they usually panic so that is when out come in to help them be referred to a hospital but cultural beliefs still play a very big role in the communities where we stay and they still hinder the ability of people to access the hospital to be screened the cancer of the cervix.” [**KII_002]**

Some African women have a belief that medical examinations should be conducted in private or darkness to avoid exposure. However, it creates a conflict when cancer screening require exposure. This practice is likely to influence the attitude of women towards cervical cancer screening. The participant noted

“… there is a culture of people thinking that any examination done to mothers should be done in the dark that is why many of the African women would prefer delivering/ giving birth while squatting because they don’t want to be exposed and yet when you are to do a cervical cancer screening you must expose the mother so there is that kind of thinking that women need a lot of privacy**.” [KII_003]**

**2.1. Effective promotional strategies**

The health facility employs multiple strategies to promote cervical cancer screening, including community dialogues, Village Health Teams (VHTs) for sensitization, and awareness campaigns through community radio and TV adverts. Additionally, informational leaflets are distributed to enhance knowledge.

“Here at the health facility we usually have community dialogues, we have Village health teams (VHT’s) they go on sensitizing people. We usually have people that come and sensitize people while using the community radio (Tiger) and sometimes adverts keep running on TV and one is able to see them for those who can be able to read them they do so. Sometime they brought us leaflets though it has taken long now we give them out although creating awareness in our community is still low and it needs to be boosted.” **[KII_002]**

“Radio and Televisions advert. Of recent we have started putting in women’s bathrooms and women’s public toilets or places of convenience*. [phone rings but he switches it off]* to enlighten them. We put stickers and also brochures to tell them about the cancer of the cervix and that has been working. And another thing is educating them through auxiliary service use. The mothers who to immunize their children others who come for family planning we are also educating them about cancer of the cervix.” **[KII_004]**

**2.2. General awareness levels**

The low awareness of cervical cancer screening is due to cultural beliefs, misconceptions about the screening process, and general ignorance, highlighting the need for more sensitization and education.

“uhmm they know less, I would not say they do not want it but because of the cultural beliefs, the nature of how women were created and also thoughts about what you are going to screen brings about dissatisfaction and this is brought about the ignorance people know less and hence they need more sensitization.” **[KII_002]**

“I think we are not yet there given a scale of 5. I would rank that we are at 3 out of 5 which is not very good.” **[KII_003]**

“Among the elite and the educated its good. Among the uneducated it’s low”. [**KII_004]**

**C- FACTORS INFLUENCING CERVICAL CANCER SCREENING**

**3.0. Social economic Impact**

Majority of the participants confirmed that cervical cancer screening is a free service at all government health facilities, making it easily accessible for women. This is further emphasized in the following quotation:

“since the screening is free actually there is no hospital so far in Uganda bit private that checks for cancer of the cervix at a cost .So I cannot say that social economic factors could be linked to a persons’ to be screened apart from the ignorance that would lead to a person to think that maybe in case I went to hospital they would tell me to buy gloves of polythene bag yet I do not have money ,we would cross out the economic factor issue because we screen for free.” **[KII_002]**

The socio-economic status of the community is not a barrier to accessing services, as all health services are free, except for transportation costs.

“That status of social economic status where I work from it is not an issue because all the services, we give are free unless when they fail to get their transport to reach the facility or even take them back. It is the same thing I told you about the infrastructure…..” **[KII_001]**

Education influences health perceptions, with educated individuals sometimes spreading misinformation, like the false claim that the HPV vaccine causes cancer. This can mislead less-educated people, who tend to trust and follow the opinions of those with more knowledge, even if it's incorrect.

“I would think for someone not having gone to school does not contribute anything to access to health care. It would be that for everyone whether learned or not we do not argue about matters to do with health but like I told you the way people perceive we have different perceptions for example one there is gentleman who is learnt, he is actually on the internet who teaches about HPV, that vaccinating HPV leads to a child contracting cancer hence people should not vaccinate against HPV then you will pity an illiterate person, how will you convince them to bring their children for vaccination meaning these that are learned usually give the illiterate information wrong information and a case scenario when a learned person says something ,the illiterate one picks up that information so fast implying that “ If a learned person has had this to say how about me who is illiterate” so they are forced to believe .So I would think when you find someone who is learned and there against something they will choose to down play it and when you find an illiterate person who loves something , they would choose to advocate for it.” **[KII_002]**

In rural areas, women’s reliance on their husbands for financial support and transportation limits their access to healthcare services, including cervical cancer screening. Additionally, the lack of economic empowerment among these women makes it difficult for them to make independent healthcare decisions.

“…. most women in rural areas depend on their husbands for anything like financial or transport so you find that even if there is a service in a health facility this woman will need transport to come to the facility and if the husband does not provide then she will not come to have the test. Additionally, most of the women are not economically empowered especially those in rural areas, the urban ones maybe but rural women are not economically empowered so it becomes hard if one is not economically empowered, so it becomes difficult to make certain decisions.” **[KII_003]**

“No! this is a mixed district; you find that there are people with very low education levels but they live in the urban and are well informed so it’s a mixture.” **[KII_003]**

“Among the elite and the educated its good. Among the uneducated it’s low.” [**KII_004]**

“Women who are rich or those that are doing well financially are more likely to screen compared to the broke ones or those that are not so doing well.” [**KII_004]**

**3.1. Religious belief influence**

Some participants are confident that women will not refuse the service based on their faith.

“ammhmu I don’t have an idea about that because what I know if I educate the woman and they understand I will give her the service. They will not stop me by telling me nurse for me am a moslem, am an Adventist, am a born again.” [**KII_001]**

“I have not heard of many apart from one religious group in Namayumba for them anything related to medical they do not accept, they do not even take treatment meaning even if you put in cervical cancer screening they will not come for it.” [**KII_003]**

Some participants highlights that some Muslim women may hesitate to undergo certain healthcare procedures, such as cervical cancer screening, due to cultural and religious beliefs that emphasize privacy.

“Moslems don’t believe one should open the legs for a health care provider unless they are pregnant. They even find it hard to open their legs when in labour. Unless when the contractions are actually very strong. So, I think Muslims are affected by religion they prefer to keep their privacy.” [**KII_004]**

And in my experience Muslims have a bad attitude with that and have been found to not want it. **[KII_004]**

Participants mentioned that cervical cancer screening faces resistance due to religious misconceptions.

“cancer yes, or no? As of me I do not second that since even in the Bible it is not allowed to tamper with our women because they are being screened of cervical cancer, actually when I talked to him about it he was able to agree that incase he gets the chance to speak to people about it, he will not hesitate to tell them the truth being that we did not know much about it. So these religious people, we are afraid of them because cancer of the cervix is a bit very sensitive of a topic and points at being vulgar sometimes you are afraid of speaking to someone hence needs to be that the person you are intending to address the issue is fully a friend of yours so that you can open up to them about issues like those.” [**KII_002]**

**3.2. Addressing religious concerns**

The participant noted that while efforts have been made to engage a small group with religious affiliations, they remain difficult to reach. Despite campaigns and discussions with their leaders, this group struggles to accept medical treatment.

“That particular one religious affiliation we have always talked to their leaders, they are a small group but they are difficult to handle because we have tried handling them for different services, we have had campaigns but it is difficult to penetrate them they really do not believe in medical treatment.” **[KII_003]**

Participant mentioned that through persistent counseling, including involving fellow Muslims to help in convincing them.

“We counsel them at some point we borrow fellow moslems to come and we get them from other departments to assist in cervical cancer screening. But after so many times of counselling them they will understand and accept to screen.” **[KII_004]**

**3.3. Role of awareness campaign**

The participant noted that women reflect on the information provided, which helps them understand the importance of screening. By highlighting that cervical cancer is linked to the HPV virus, often transmitted by their husbands, women are more likely to recognize the need for screening and take action.

“They first reflect from where they come from then think about what we have told them then she analyses that she needs to get screened. The information we usually tell them is that this virus is that women are not shy to get screened and that it is brought into you by your husband. Now if the woman understands that my husband has many wives like five or four deep down it becomes easy for her to decide to go and get screened because her husband could have got it from her first wife**.” [KII_001]**

Participant mentioned that since training in 2002, awareness has significantly increased, and screening services are now more accessible. While some remain hesitant, efforts are paying off, with NGOs now actively addressing cervical cancer in response to growing awareness.

“We were taken through training around 2002, this is because it was to a point of 0.0:1of mother comes for cervical cancer screening. Secondly the services were not readily available in the centers were am I leading you unto that because there was no awareness people never used to come for screening but now that awareness has been boosted, we can now be able to screen at least people are aware and it depends on the person not to be willing however for those that choose not to understand it stays that way but now most of the people know and the screening services are readily available. Therefore our efforts are starting to pay off slowly, for that case that is why some Non-Government Organizations (NGO’s) are now coming into existence in Uganda after them getting aware that this sort of cancer is in existence.” **[KII_002]**

“They play a big role. They are like the guardian angel of the program. They influence women’s perception and they encourage women to actively participate to seek cervical cancer screening services.” **[KII_004**]

**3.4. Perceived health and volubility**

Fear of having cervix removed or concerns about not recovering if diagnosed can lead several women to refuse screening. Such beliefs, rooted in personal perceptions, can either encourage or discourage women from getting screened.

“Yes, if a woman perceives that they do not feel like they should not be screened of the cancer of the cervix, with perception of fear that their cervix could be removed they can choose to refuse. Another one can think that in case they find that I have this cancer I will not get well but will rather remain sick of it so they choose to be refuse to be screened because it is their thinking is their golden attribute so that kind of perception could force them to allow to be screened or not.” **[KII_002]**

Participants discussed that a woman who has experienced serious health issues is more likely to prioritize her health and take cervical cancer screening seriously, compared to one who has never faced major health challenges.

“….. however, a woman who has been through hell and back about their health is likely to take their health serious than the one who has not been ill. So, I think a woman who has suffered a big health condition will take their life more serious to have cervical cancer screening more serious more than one who has not.” **[KII_004]**

**3.5.** **Social network and family influence**

Women who are not empowered often need permission to access healthcare, whereas empowered women can make decisions independently. This disparity affects service utilization, even among mothers.

“As I said women who are not empowered you can’t make a decision to access a service. You find that there are other women who also have children but still carry those babies and they come for a service so I think it is their empowerment which is a problem. Those that are empowered easily access a service and those that are not empowered need clearance to go for a service.” **[KII_003]**

Stay-at-home mothers often struggle to access healthcare due to childcare responsibilities. Without older children to help, they may miss medical visits, limiting their access to essential health care services like cervical cancer screening.

“… taking care of children for example for the stay home mothers sometimes they do not have people to stay with the children as soon as they leave, therefore unless if they have been able to have those children that are older in a home those that can stop from going to school on a particular day so that the parent can go to the health facility so in other wards staying home to take care of the children leads them to be stopped to get to the health centers to access support.” **[KII_002]**

**3.6. Fear of diagnosis**

Fear of harm, pain, and embarrassment discourages women from cervical cancer screening. Concerns about the equipment and discomfort, especially with cervicitis, create hesitation. Clear education and reassurance are key to overcoming these barriers.

“Yes, these methods hinder them this is because do not want to tampered with, thinking about the equipment used to screen how it could end up damaging them so it becomes hard. Actually, one needs to persuade people that it has no harm it can cause to be able to tempt them into screening at the time when the explanations about the equipment are done, one can now move forward but with the other factors connected such as pain, sometimes they have cervicitis when you attach the equipment they develop some sort of pain in the process one can scream if there other people out this ends up scaring them [participant laughs out loud].” **[KII_002]**

Fear and embarrassment deter many women from cervical cancer screening. Concerns about sample collection and anxiety over a positive result often drive them away, despite attending health education sessions.

“Actually, it drives off majority of the women. They would come for the health education but when they are told that the samples will be picked from the private parts, they will shy away maybe because they feel they are not clean enough that day, they have different reasons, and then the fear, it’s like an HIV test it is easy to say I might go there; during health education they are usually told what causes this and that and then someone will check through their lives and they say I think I am this – how about if I got the results positive? So it chases them away.” **[KII_003]**

**3.7. Past women healthcare experiences**

Positive experiences encourage women to repeat screening, while negative experiences deter majority of them.

“Those that have a good experience with screening are likely to come back and screen whenever recommended. Those that have had a bad experience end up not taking it up again. But usually, I think if someone has enhancing factors that stimulated the first screening if they sustain she will come back for more.” **[KII_004]**

Participants discussed that quality customer care in healthcare is crucial. While some health workers provide excellent service, a few may be rude, discouraging patients.

“It depends on the customer care that is given to them from individual facilities, I cannot deny that we have health workers who have very good customer care then there are those that are rude, a few of them so if they land on a rude one I think it will really drive them away so it is our customer care that is very demanding.” **[KII_003]**

**3.8. Logistical barriers**

Women often miss cervical cancer screening due to time constraints, household responsibilities, and lack of support at home. Since the procedure requires a sterile environment, it cannot be done at home, leaving many unable to access the service.

“They do not get time to come to the hospital and for us, we cannot take the service at their home. This is because it is a sterile procedure. Meaning we have to do this procedure in a very clean place so we cannot go at her home. So if they have a lot of work at home, and children disturbing her even the husband who needs her attention and she gets no time to come to hospital. Then she will miss the service.” **[KII_001]**

Financial and childcare challenges prevent many women from accessing cervical cancer screening. Never the less, the cost of transportation may be unaffordable.

“Yes, for example if a woman is coming from a village called Kamusogonya, yet they have to move to close by village in Butooro, within that distance it takes about 10,00 Uganda shillings but it is even hard for some of them to have that money. At a time they would expect to get the money sometimes it is too late to be worked on.” **[KII_002]**

Distance, time constraints, and transportation issues significantly impact women's access to cervical cancer screening. Women living farther from health facilities are less likely to get screened. Even during medical camps, when demand exceeds capacity, many women are unable to be screened due to limited resources.

“Those whenever at a lower side they are affected negatively and it will impact women’s up-take because a woman who stays 10kilometers away from the screening services is less likely to do cervical cancer screening compared to one staying a stone throw away. But also, time constraints, transportation sometimes we go for medical camps and maybe we have targeted 100 women to be screened. And we get 250 what do we do with the rest? They don’t screen and get discouraged to come back the next time. Which means it negatively impacts.” **[KII_004]**

**Strategies to overcome barriers**

To improve access, outreach programs have been introduced, bringing screening services closer to communities. While services can't be provided at home, local council areas are used as gathering points where women can easily access screening services.

“We now started to do out reaches in this we now brought the services closer to where they stay although we cannot bring the services to their homes. For one who would want to access the service we usually gather at a local council area find a place where we can screen from so whoever wants the service, they can access the service there.” **[KII_002]**

The government built smaller health centers, providing screening services twice a week with necessary supplies like acetic acid. Additionally, the referral system has been strengthened, with Village Health Team members identifying women in need of screening

“The government has been able to build small health centers such as health centers 2 and now the service is available at least twice a week where women can be ably screened and also some items such as acetic acid are always available. The other thing we beefed up the referral system, when we find a woman in the community that needs screening we usually invest in some of our finances to facilitate the woman to be able to come to the health center for checkup that facilitation carters for both bringing them and taking them back home this is done so that they do not claim they failed to get the service for as long as the Village Health Team member is the one that identifies this case we organize and make sure they can be brought to the facility.” **[KII_002]**

Women’s health empowerment is central to the strategy, with health education targeting both women and men. Radio talk shows and HPV vaccination programs, backed by education on cervical cancer, help increase community awareness and participation.

“We do a lot of women empowerments, women must be empowered to take care of their own health. It is a strategy that we are using in many of the maternal child health services. Additionally, the health education that we give does not only address women it is a mixed community, so we address both women and men then decisions are made and then 3, the radio talk shows that we are using also is a good source for us to have a good number of women turning up and finally we have an immunization program especially around HPV. Before we give HPV, we try our best to educate communities about cervical cancer and why we are giving the young girls the HPV vaccine.” **[KII_003]**

To make women feel more comfortable, female doctors, nurses, and midwives are prioritized in the healthcare field. Women are generally more at ease being screened by fellow women

“For some cases were we have female doctors, nurses and midwives we use those I think we have deliberately recruited many women into that field as compared to males. And women are likely to be are comfortable around women or screened by fellow women than males. And two we still continue educating women that the benefits out way the risks. Today your saying ‘you are kusitamilaling basawo’ because you have a choice. Tomorrow when you have the real cancer of the cervix you will beg to ‘kusitamila musawo so that they can help so that they can treat your cancer of the cervix. So, how about you do it when it’s a bit earlier.” **[KII_004]**

Participants discussed that staff should remain dedicated while seeking support from donors, partners, and organizations like the cancer institute.

“I encourage the few available staff to be steadfast. Lobbing from donors and well-wishers and partners for example the cancer institute is giving us quite a lot of materials in XX hospital. So I also encourage women the ground people, the leaders and the health care systems to help bail us out as the political will comes. For example, the director of XX of recent hired many staff on contract and those people are at least helping by giving a hand to relieve the burden of work with those already there.” **[KII_004]**
